# Multimodal Single‐Cell Analyses Outline the Immune Microenvironment and Therapeutic Effectors of Interstitial Cystitis/Bladder Pain Syndrome

**DOI:** 10.1002/advs.202106063

**Published:** 2022-04-25

**Authors:** Fei Su, Wei Zhang, Lingfeng Meng, Wei Zhang, Xiaodong Liu, Xiaorui Liu, Meng Chen, Yaoguang Zhang, Fei Xiao

**Affiliations:** ^1^ Clinical Biobank Beijing Hospital National Center of Gerontology Institute of Geriatric Medicine Chinese Academy of Medical Sciences Beijing 100730 P. R. China; ^2^ The Key Laboratory of Geriatrics Beijing Hospital National Center of Gerontology Institute of Geriatric Medicine Chinese Academy of Medical Sciences Beijing 100730 P. R. China; ^3^ Department of Pathology Beijing Hospital National Center of Gerontology Institute of Geriatric Medicine Chinese Academy of Medical Sciences Beijing 100730 P. R. China; ^4^ Department of Urology Beijing Hospital National Center of Gerontology Institute of Geriatric Medicine Chinese Academy of Medical Sciences Beijing 100730 P. R. China; ^5^ Shanghai Key Laboratory of Embryo Original Diseases The International Peace Maternity and Child Health Hospital School of Medicine Shanghai Jiao Tong University Shanghai 200030 P. R. China; ^6^ Key Laboratory for National Cancer Big Data Analysis and Implement National Cancer Data Center National Cancer Center/National Clinical Research Center for Cancer/Cancer Hospital Chinese Academy of Medical Sciences and Peking Union Medical College Beijing 100021 P. R. China

**Keywords:** bladder, image mass cytometry, inflammation, interstitial cystitis/bladder pain syndrome, single‐cell RNA sequencing

## Abstract

Interstitial cystitis/bladder pain syndrome (IC/BPS) has a significant impact on quality of life, but the etiopathogenesis remains largely unknown. The bladder microenvironment of patients with IC/BPS to obtain biological evidence supporting diagnosis and novel therapy is systematically characterized. Single‐cell RNA sequencing (scRNA‐seq) and image mass cytometry (IMC) are applied to bladder biopsies of the IC/BPS cohort. A total of 42 distinct cell clusters are identified from different groups. The increased hyperactivated Th1‐biased response, but not Th2‐biased response, and decreased immunosuppressive Treg are elucidated in the bladder microenvironment of non‐Hunner‐type IC (NHIC)/Hunner‐type IC (HIC). M2/M2‐like macrophage extends in the HIC and M1‐like macrophage extends in NHIC, all of which secrete a range of chemokines with different pattern. The pro‐inflammatory mediators, TNF‐α, produced by tissue‐resident macrophages and IL6, by the inflammatory fibroblasts are identified as key mediators of IC/BPS pathogenesis. Additionally, a regulatory network between different cell types is observed as a shift from structural cell communication in unaffected normal bladder to a Macrophage‐Endothelial‐dominated interactome in NHIC/HIC. The results demonstrate the high heterogeneity in NHIC/HIC, and provide an essential resource for diagnosis, and treatment of IC/BPS in the future by highlighting the importance of the microenvironment of bladder mucosa.

## Graphical abstract

Single‐cell RNA sequencing analyses and imaging mass cytometry reveal increased hyperactivated Th1‐biased response and decreased immunosuppressive Treg in the IC/BPS. Pro‐inflammatory cytokines and TNF‐α from macrophages and IL6 from fibroblasts are identified as key mediators of IC/BPS pathogenesis. This study provides an essential resource for diagnosis and treatment of IC/BPS by highlighting the importance of the microenvironment of bladder mucosa.

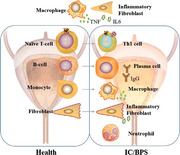

## 1 Introduction

Interstitial cystitis/bladder pain syndrome (IC/BPS), a chronic bladder disease with an increasing incidence, affects millions of individuals across the world.^[^ ^1^ ^]^ According to the International Society for the Study of Bladder Pain Syndrome (known as ESSIC) guidelines, IC/BPS can be classified into two main pathological subtypes based on endoscopic findings: IC/BPS with Hunner lesions (HIC) and with glomerulations but no Hunner lesions (NHIC).^[^ ^1^, ^2^ ^]^ The etiology of IC/BPS is still unknown.^[^ ^3^ ^]^ The human bladder mucosa is composed of a single layer of urothelial cells and multiple types of immune cells. The urothelial barrier prevents solutes and bacteria from entering the urothelium to activate immune cells. Changes in a variety of immune cells including plasma cells^[^ ^4^ ^]^ and mast cells^[^ ^5^ ^]^ have been observed and are believed to be associated with IC/BPS development. Pro‐inflammatory cytokines were also identified in the urine of IC/BPS patients.^[^ ^6^ ^]^ Thus, immunologic dysfunction may be one of the possible causes of IC/BPS.^[^ ^7^ ^]^ Compositional and functional alterations of the structural cells (including epithelial cells and fibroblasts) can be seen in the bladder tissue of patients with IC/BPS.^[^ ^8^ ^]^ On the other side, IC/BPS is a highly heterogeneous disease. The two pathological subtypes, NHIC/HIC, show different clinical features in many aspects.^[^ ^3^ ^]^ It is unclear whether HIC represents a separate disease process compared to NHIC. Determining the abnormal pathways in NHIC/HIC could help to identify whether HIC is part of the NHIC or a unique disease.

Several treatments are available for IC/BPS, including steroid injections, immunosuppressive drugs, and novel medications such as anti‐TNF*α* agents are under development.^[^ ^9^ ^]^ However, the currently available treatments often fail or become less effective over time. Defining key cellular subsets and their activation states in IC/BPS is also a critical step for developing novel diagnosis methods and defining new therapeutic targets.

Single‐cell RNA sequencing (scRNA) and image mass cytometry (IMC) techniques offer an opportunity to identify disease‐associated cell subsets in human tissues at high resolution in an unbiased fashion.^[^ ^10^ ^]^ In our current study, we performed scRNA and IMC to profile cells from five patients with NHIC/HIC and two from unaffected control (UC) bladder tissues to explore the molecular mechanisms of IC/BPS. It was found that the common pathogenesis features of IC/BPS included inflammatory fibroblasts, activated CD4^+^ T cells, neutrophils migration and activation of autoimmune‐associated B cells. Also, we conducted a comprehensive transcriptomic analysis for novel candidate disease biomarkers and dissected the intercellular crosstalk between structural and inflammatory cells, which might shed a light on the treatment strategies for IC/BPS.

## 2 Results

### 2.1 Cohort Characteristics and Single‐Cell Profiling Results of Bladder Microenvironment

**Figure 1:**
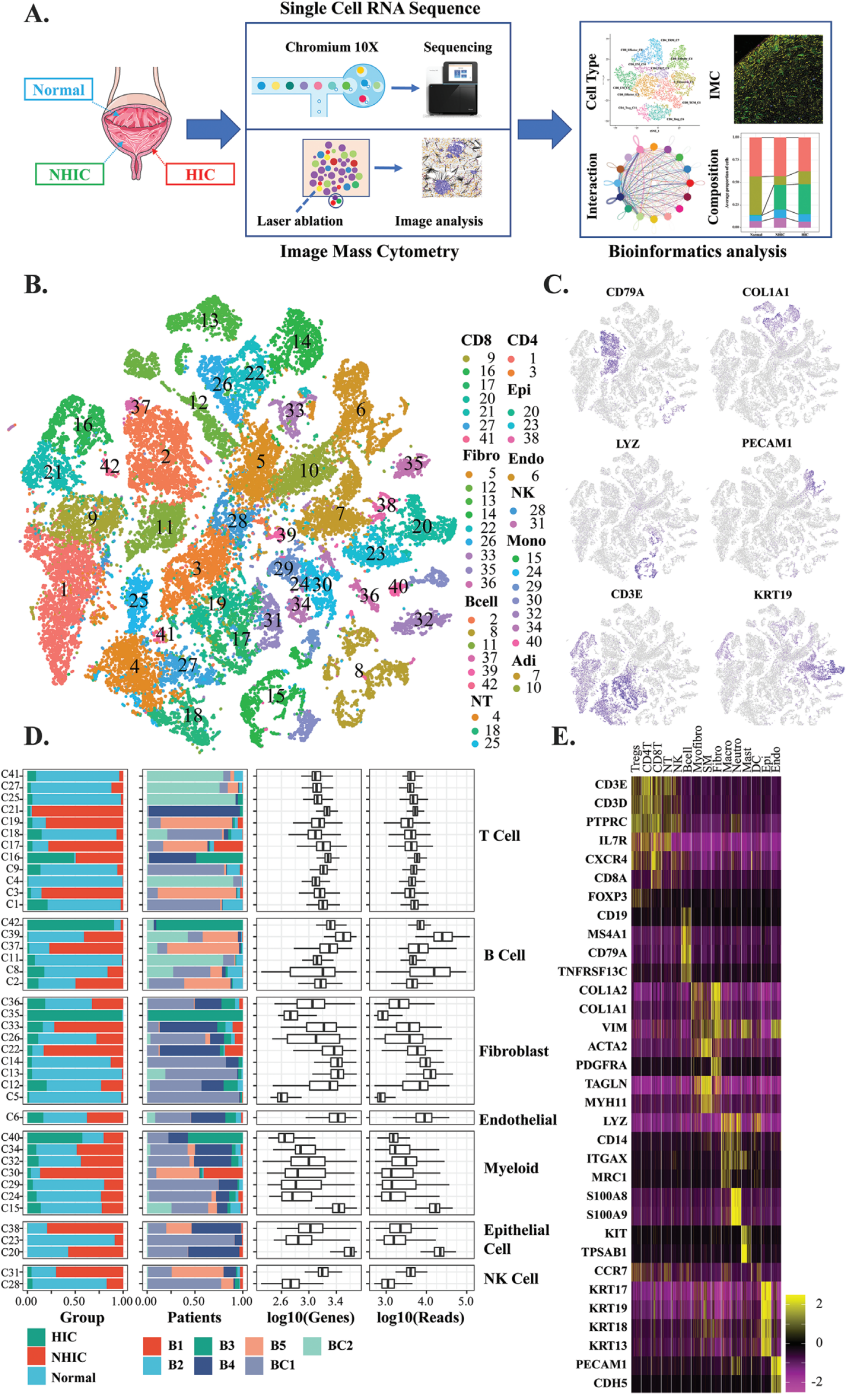
A) Schematic diagram of scRNA‐seq analysis workflow. Bladder tissues were dissociated into single cells and sequenced using 10X Genomics platform. Additional samples were used to validate the results of scRNA‐seq by IMC platform. B) *t*‐SNE plots of cells from the 7 samples profiled in this study, with each cell color coded to indicate the associated cell types. C) *t*‐SNE plots showing the canonical marker gene expression of myeloid cells (LYZ), T cells (CD3E), B cells (CD79A), Fibroblast (COL1A1), Endothelial cells (PECAM1), and Epithelial (KRT19). D) For the 42 cell types (left to right): the fraction of cells originating from each groups, samples, box plots of the number of genes and reads (with the box plot center, box, whiskers, and points corresponding to the median, interquartile range, 1.5 × interquartile range, and outliers, respectively). E) Heatmap of key cell‐type markers across the cellular compartments. Heatmap visualization color‐coding the UMI counts per single cells (stacked columns) for selected marker genes (rows). Visualized are 500 randomly selected cells per cluster.

We performed droplet‐based scRNA‐seq to study the transcriptomic profiles of bladder mucosa samples from 5 NHIC/HIC patients and 2 patients with non‐muscle invasive bladder cancer as UC (**Figure** **1**A). The clinical characteristics of eligible studies are detailed in Table S1 (Supporting Information). All patients were treatment‐naive females, and patients with an acute bacterial infection were excluded. None of the patients had a history of lymphatic disease. HIC patients had significantly higher International Prostate Symptom Score and smaller bladder capacity than those with NHIC and UC (Table S1, Supporting Information). More severe symptoms tended to manifest in HIC patients than in NHIC patients. After quality filtering, 304 million unique transcripts were obtained from 44490 cells, in which over 200 genes were detected as positively expressed (Table S2, Supporting Information). The method described by Gervaise et al.^[^ ^11^ ^]^ was applied to detect stressed cells. About 10% of the total cells were removed due to a high stress signature (Figure S1A,B, Supporting Information). To further ensure that our analysis was restricted to non‐malignant bladder cells, we inferred single‐cell DNA copy number alteration (CNA) profiles. scRNA‐seq data from NHIC/HIC were used as a normal reference and no cells were predicted as malignant cells (Figure S1C, Supporting Information). Ribosomal and mitochondrial genes were removed from the variable genes used for principal component analyses (PCA) and clustering. Graph‐based clustering was performed to partition the 40972 high‐quality cells into 42 distinct cellular clusters, which was shown with *t*‐distributed stochastic neighbor embedding (*t*‐SNE) plot (Figure 1B). We also calculated the ROGUE score to determine the purity of cell clusters, most clusters were highly pure, with the majority having a score over 0.8 (1.0 indicating 100% purity, Figure S2A, Supporting Information). According to the expression of the canonical markers (Figure 1C–E; Table S3, Supporting Information)^[^ ^12^ ^]^ and automated cell‐type classification (Figure S2B, Supporting Information),^[^ ^13^ ^]^ each cluster was further assigned into specific cell subpopulations, including *CD4* ^+^ T cells (marked with *CD3D* ^+^ and *CD4* ^+^; mean frequency: 15.8%), CD8^+^ T cells (*CD3D* ^+^/*CD8A* ^+^; 21.4%), myeloid cells (*CD14* ^+^/*LYZ* ^+^; 11.0%), B cells (*CD19* ^+^/*MS4A1* ^+^; 14.8%), endothelial cells (*CDH5* ^+^/*PECAM1* ^+^; 4%), fibroblasts (*COL1A1* ^+^ / *COL1A2* ^+^; 18.5%), and epithelial cells (*KRT18* ^+^/ *KRT19* ^+^; 4.7%). Two cell clusters expressed canonical markers from different cell types, and they were considered doublets (Table S3, Supporting Information). Nerve cells were not detected, probably due to technical limitations. In order to compare the composition of the cell types among conditions, we normalized the cell counts and found some cell subclusters were highly condition‐specific (Figure 1D).

### 2.2 Dysfunctional *CD4* ^+^ T Cells Were Significantly Increased in the IC Samples

**Figure 2:**
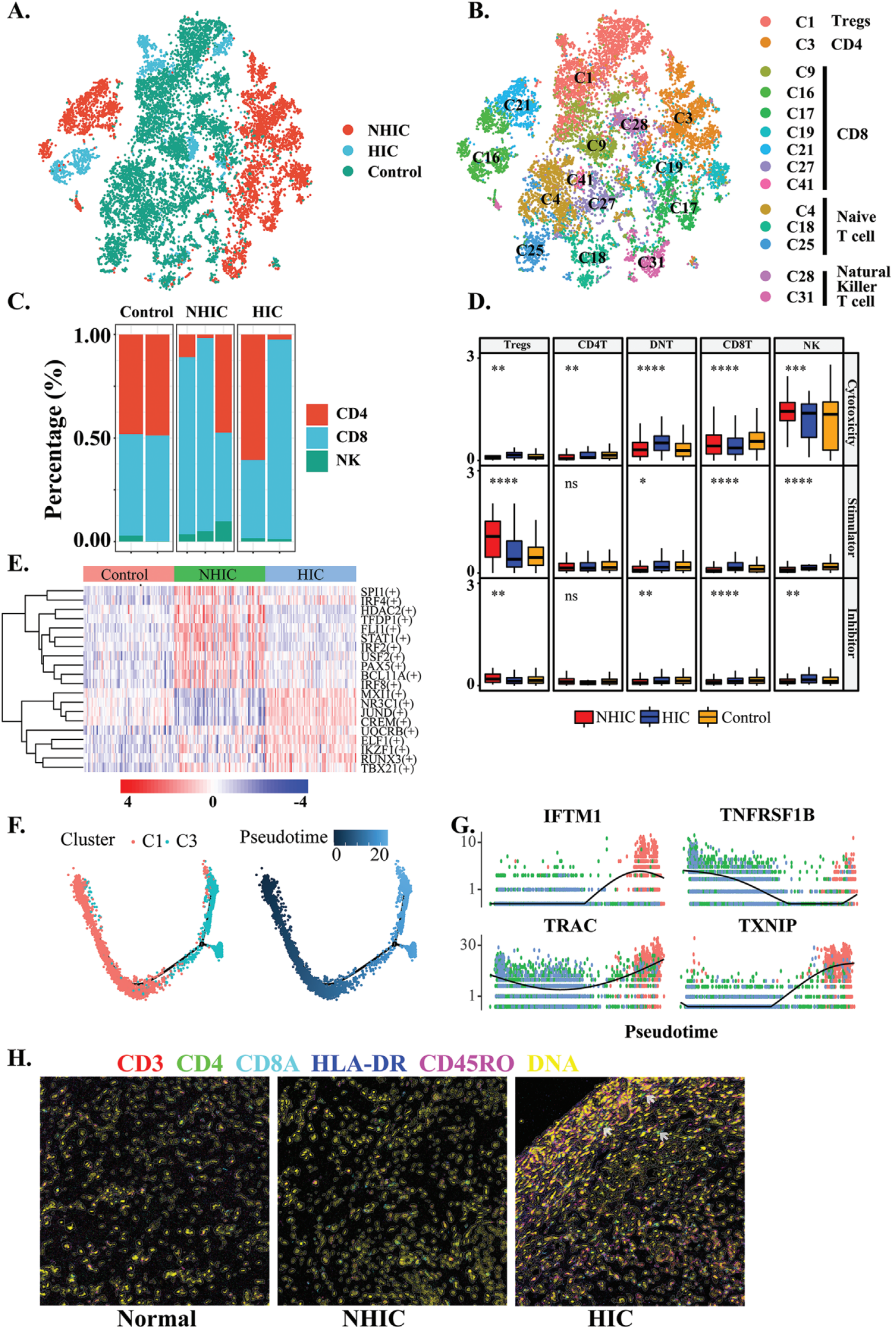
A) *t*‐SNE plot of T/NK cells colored by different groups. B) *t*‐SNE plot showing 14 clusters of 15 888 T/NK cells colored by cell types. C) Percentage of CD4^+^ T cells, CD8^+^ T cells, and NK cells in the different samples and groups. D) Boxplot of cytotoxicity, co‐stimulatory and co‐inhibitory scores defined by related genes for the three groups. Significance was determined by Student's *t*‐test. NS, not significant (*p* > 0.05); *p ≤ 0.05; **p ≤ 0.05; ***p ≤ 0.05; ****p ≤ 0.05; E) Heatmap showing the activity of TFs in each T cell subtype. The TF activity is scored using AUCell. F) Developmental trajectory of *CD4* ^+^ T cells (*n*  =  4787) inferred by analysis with Monocle2, which colored by different cell cluster (left) and pseudotime (right). G) Top genes calculated by Monocle2 were shown as dot plots displayed as expression level over pseudotime. Red points stand for HIC. Green stands for NHIC. Blue stands for unaffected normal. H) False‐color images for CD4 (red), (CD8A) green, FOXP3 (blue), HLA‐DR (cyan), CD3 (magenta), and DNA (yellow) antibody stains are shown for IMC. Scale bars, 150 mm.

The immune response of T cells was heavily skewed toward Th2 immune responses during other diseases.^[^ ^14^ ^]^ However, the role of T cells in the IC/BPS was largely unknown. We first explored the intrinsic structure and functional subtypes of the T/NK cell populations (Figure S3A, Supporting Information). With 15888 cells detected, T cells (37.2%) were the main immune cell population and showed with high heterogeneity, which were composed of six *CD8* ^+^ T cell clusters, *CD4* ^+^ type 1 T helper cells (Th1; *TRAC* ^+^, *FOXP3* ^−^, *CD4* ^+^), regulatory T cells (Tregs; *FOXP3* ^+^), two NK cells (*NKG7* ^+^) and three double negative T (DNT) cells (Table S3, Supporting Information; **Figure** **2**A,B). According to the distribution of cell types, CD4^+^/CD8^+^ ratio (normalized cell number of CD4^+^ T cell/CD8^+^ T cell) remarkably decreased in patients with NHIC (0.417)/HIC (0.814) than in UC (0.967; Figure 2C). We identified six *CD8* ^+^ T cell clusters characterized by distinct expression patterns of effector molecule genes (Figure S3B, Supporting Information), and these populations were defined as *GZMK* ^+^ (C9 and C19), *GZMK* ^+^ *GZMB* ^+^ (C17 and C27), and *GZMB* ^+^ cytotoxic T lymphocytes (C21). In addition, we also identified a *CD8* ^+^ T population (C16) which expressed *IL1B* suggestive of an effector phenotype (Figure S3B, Supporting Information). As shown in Figure 2D, the NK cells showed higher cytotoxicity scores than the other cells. Meanwhile, Tregs from HIC/NHIC showed highest co‐stimulatory scores.^[^ ^15^ ^]^ Pathway activity analysis among different groups with GSVA and GO analysis suggested that genes related to immune activation were consistently enriched in the T cells from HIC/NHIC (Figure S3C,D, Supporting Information). Besides, Th1 cells were strongly activated with T cell receptor signaling with highly expressed *TRAC*, *TRBC1*, and *TRBC2*, suggesting Th1 might be triggered by donor antigen on antigen presenting cells (APCs). IMC confirmed the presence of activated *CD4* ^+^ cells close to urothelium, consistent with the results of scRNA‐seq in the HIC group (Figure 2H and Figure S4, Supporting Information). IMC also showed that there were more activated *CD8* ^+^ T cells in the NHIC/HIC groups than UC. A validation dataset (GEO: GSE11783) for gene expression from UC and NHIC/HIC patients was used to calculate the signature scores from bulk samples (Figure S5, Supporting Information).^[^ ^16^ ^]^ SCENIC for overrepresented transcript factors (TFs) and their putative target genes across conditions revealed that genes of IRF family (IRF2, IRF4, and IRF8), a group of transcriptional regulators of IFNs and IFN‐inducible genes involved in the control of T cell differentiation during inflammation, were the most specific regulons associated with the NHIC group (Figure 2E). Compared to UC, the NHIC/HIC groups had highly activated *TBX21* and *RUNX3*, which promoted the conversion of Tregs to Th1 cells.^[^ ^17^ ^]^ With trajectory analysis of 4787 *CD4* ^+^ cells with Monocle2, we observed the developmental trajectories from Tregs cells at the initial state to activated *CD4* ^+^ cells at the terminal state (Figure 2F and Figure S6, Supporting Information). In support of the plotted pseudo‐timeline order, our data revealed groups of genes that showed differential expression along each axis, which correlated well with immune‐regulating genes, such as *TXNIP* and *IFITM1*, important host effector molecules of the type I interferon response (Figure 2G and Table S4, Supporting Information). Overall, the results provide a landscape of T/NK cells in the bladder microenvironment, suggesting that the Th1‐biased response was primary in the NHIC/HIC.

### 2.3 Activated Macrophages Enhanced Neutrophil Recruitment into the Bladder Mucosa

**Figure 3:**
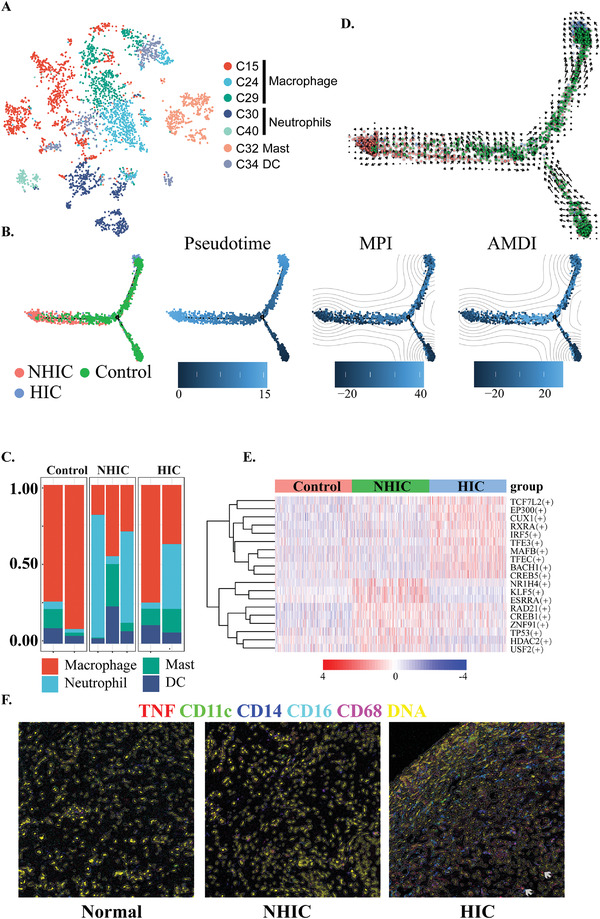
Pseudotime analysis of Macrophage cells by using RNA velocity reconstructed the developmental lineages. A) *t*‐SNE plot showing 7 clusters of 4489 myeloid cells from different groups, which colored by different cell types. B) Trajectory of differentiation from monocyte into tissue resident macrophages predicted by monocle 2 colored by different groups (left), pseudotime (middle 1), MPI (middle 2), and AMDI (right) scores. Pseudotime of TRMs was colored in a gradient from dark to light blue, and the start of pseudotime was indicated. TRMs pseudo‐timeline, colored according to the corresponding MPI and AMDI scores; dark blue indicates greater pro‐inflammatory/maturity. C) Velocity field projected onto *t*‐SNE plot of the cell states of TRMs. D) Percentages of cellular lineages in each condition included in the scRNA‐seq analysis. For each sample, a bar graph depicts the percentage of cells in clusters associated with each lineage. E) Heatmap of the area under the curve (AUC) scores of TF motifs estimated per cell by SCENIC. Shown are top five differentially activated motifs in the NHIC and HIC groups, respectively. F) False‐color images for TNF‐α (red), CD11c (green), CD14 (blue), CD16 (cyan), CD68 (magenta), and DNA (yellow) antibody stains are shown for IMC. Consecutive tissue sections of the same region of the tumor were analyzed. Scale bars, 150 mm. White arrows in the HIC arrows indicate the CD68^+^ TRMs.

Monocytes/macrophages play pivotal roles in the host innate immunity and serve as a first line of defense in bladder mycobacterial infection, and also modulate T cell activation or limit differentiation of memory T cells.^[^ ^18^ ^]^ A total of 4489 myeloid cells were identified and categorized into 7 transcriptionally distinct subsets (**Figure** **3**A and Figure S7A, Supporting Information): aged neutrophils (C40; *S100A9* ^+^/*S100A8* ^+^/*CXCR4* ^+^), naïve neutrophils (C30; *S100A9* ^+^/*S100A8* ^+^/*SELL* ^+^), mast cells (*KIT* ^+^/*TPSAB1* ^+^), 3 tissue‐resident macrophages (TRMs; *CD68* ^+^/*MRC1* ^+^), and DCs (*HLA‐DRA* ^+^/*CCR7* ^+^). The pro‐inflammatory neutrophils (C40 and C30) were mainly present in the HIC (14.8%) and NHIC (72.9%). Aged neutrophils (C40) highly expressed pro‐inflammatory cytokine oncostatin M (OSM, Figure S7B, Supporting Information), which play a critical role in the induction of inflammation and the modulation of extracellular matrix (ECM).^[^ ^19^ ^]^ Based on scRNA‐seq, TRMs were divided into three subsets (Figure S7C, Supporting Information): *CLEC10A* ^+^ TRMs (C15) expressed markers of DCs (*CD1C* and *CLEC10A*); *C1QA* ^+^ TRMs (C24) highly expressed complement pathway activators (*C1QA*/*C1QB*) and markers of M2 macrophage (*SELENOP*);^[^ ^20^ ^]^ and *IL1B* ^+^ TRMs (C29) expressed *IL1B*, *CCL3*, and *CXCL3*. TRMs from C15 and C29 were highly expressed the pro‐inflammatory cytokine, TNF‐α, that mediate tissue pathology in rheumatoid arthritis^[^ ^21^ ^]^ and IBD.^[^ ^10b^ ^]^ We indeed observed significant enrichment of the TNF‐alpha signaling via nuclear factor‐*κ*B (NF‐*κ*B) pathway in these two groups. Moreover, IMC analysis also identified that there were more TRMs with high levels of TNF in the NHIC/HIC than UC (Figure 3F). In addition to increased *TNF* expression, up‐regulated NLRP3 were also seen in C15 and C29, which is the main component of inflammasome. The assembly of NLRP3 inflammasome can promote maturation and secretion of IL‐1*β* and IL‐18, which drive inflammatory response.

GO analysis showed that TRMs mainly involved inflammation‐related terms such as granulocyte chemotaxis, antigen processing, neutrophil degranulation, suggesting TRMs might play a critical role in the pathogenesis of IC/BPS (Figure S7D, Supporting Information). To understand the development and polarization of TRMs, we applied MacSpectrum to calculate the MPI and AMDI scores of each TRM cell. M1‐like/pre‐M1 cells related to pro‐inflammatory were expanded in the NHIC/HIC samples (Figure S8, Supporting Information) and the results showed that macrophages from different conditions had distinct MPI distribution (Figure S8A, Supporting Information) (UC: mean: −5.01; 95% CI: −5.92 to −4.11; NHIC patients: mean: −2.89; 95% CI: −4.47 to −1.30; and HIC patients: mean: 2.91; 95% CI: 1.01–4.80), suggestive of M1 polarity in the NHIC/HIC. The distribution of AMDI (Figure S8B, Supporting Information) (UC: mean: −8.66; 95% CI: −9.76 to −7.55; NHIC patients: mean: −4.08; 95% CI: −6.02 to −2.15 and HIC patients: mean: 1.39; 95% CI: −1.09–3.87) was associated with a mature phenotype of TRMs.

Pseudotime analysis of the relationships among the cell clusters showed a branching differentiation trajectory with two terminally clusters (States 1 and 3; Figure 3B) stemming from the same precursor cluster (State 2). According to cell types, these three branches were composed of different cells from M1‐like/pre‐M1 (State 1), M0 (State 2), and a mixture of M1/M2 (State 3; Figure S8, Supporting Information). RNA velocity analysis confirmed the pseudotime results and continued polarization of macrophage from M0 to M1‐like (Figure 3C). The 50 DEGs was clustered via a pseudo‐temporal expression pattern, as shown by the heat map (Figure S9, Supporting Information). Genes affecting cell developmental trajectory were divided into two clusters: State 1, which mainly from NHIC, consisted of genes highly expressed the genes related to inflammation and chemokines, such as *CCL2*, *CXCL8*, *CXCL3*, and antigen presentation (*HLA‐DRB5*) and *NRP2*; State 3, which includes *IFITM2*, *TXNIP*, *PTPRC*, and *JUNB*, was enriched in the infection response (Figure 3D). SCENIC showed *EP300* and *MAFB*, which regulate the late stages of granulocyte differentiation, were most significantly in the HIC (Figure 3E). Other genes, such as *ELF2*, *ELF4*, and *BCLAF1* also showed a similar pattern of regulon activation in the NHIC/HIC groups. Thus, infiltration of hyper‐inflammatory macrophages is a hallmark of IC/BPS, which could be targeted by TNF‐α inhibitors such as Adalimumab.

### 2.4 Fibroblasts from IC/BPS Subjects Were Enriched with Inflammatory

**Figure 4:**
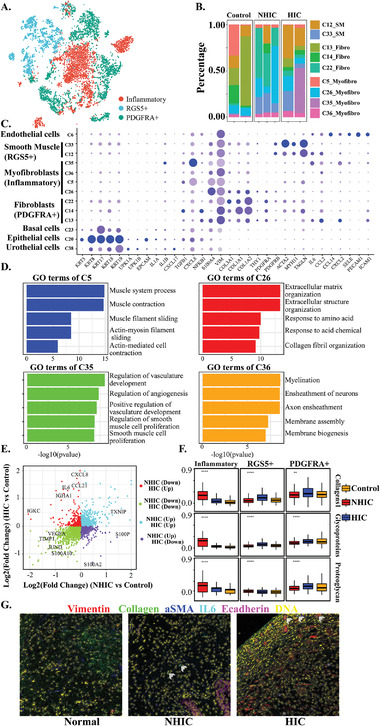
A) *t*‐SNE plot showing 7578 fibroblasts from different groups indicated by colors. B) Percentages of cellular lineages in each condition included in the scRNA‐seq analysis. For each sample, a bar graph depicts the percentage of cells in clusters associated with each lineage. C) Dot plot shows the expression level of key functional genes and canonical markers of fibroblast cells. D) GO analysis of DEGs in distinct fibroblast subclusters. E) Scatter plots of fold change of fibroblasts from conditions, *x*‐axis stands for log2 transferred fold change between NHIC and Control, *y*‐axis stands for log2 transferred fold change between HIC and Control. F) Boxplot of collagens, glycoproteins and proteoglycans scores defined by related genes for the three conditions. Significance was determined by Student's *t*‐test. NS, not significant (*p* > 0.05); *p ≤ 0.05; **p ≤ 0.05; ***p ≤ 0.05; ****p ≤ 0.05; G) False‐color images for SMA (blue), IL6 (cyan), E‐cadherin (magenta), Collagen (green), Vimentin (red), and DNA (yellow) antibody stains are shown for IMC. Consecutive tissue sections of the same region of the tumor were analyzed. Scale bars, 150 mm. White arrows indicate the IL6+ fibroblasts.

Fibroblasts were clustered into three different subtypes (**Figure** **4**A): a) PDGFRA^+^ fibroblasts, which exhibit strong expression of major collagen genes including *COL1A1*, *COL1A2*, and *COL3A1*; b) RGS5^+^ fibroblasts, which have characteristics similar to vascular smooth muscle cells with expressions of *ACTA2*, *TAGLN*, and *MTY11*; and c) inflammatory fibroblasts, which strongly express chemokines including *CCL2*, *CXCL2*, CXCL8, and *IL6*, suggesting these cells were in an active cytokine‐producing state and similar to cancer‐associated fibroblasts described in many studies^[^ ^22^ ^]^ (Figure 4E). Moreover, IMC verified that IL6 expression was mainly localized with inflammatory fibroblasts, in accordance with our scRNA‐seq findings (Figure 4G). Importantly, IL6, a drug target and involved in the pathogenesis of many diseases, was highly expressed in the samples from HIC, which provide potential targets for inhibiting or reversing the immune dysfunction of IC/BPS. GO analysis on the top 50 DEGs of every cluster showed that C35, which was mainly from HIC group (98.7%, Figure 4B), highly expressed the pro‐inflammatory cytokines and was enriched in biological activities for vascular development and angiogenesis, whereas C26 expressed genes enriched in the ECM and epithelial to mesenchymal transition (EMT) (Figure 4D). Calculation of ECM expression score showed inflammatory fibroblasts exhibited the highest ECM expression (Figure 4F).

### 2.5 Tissue‐Resident Memory B Cells and IgG^+^ Plasma Cells Were Enriched in NHIC/HIC

**Figure 5:**
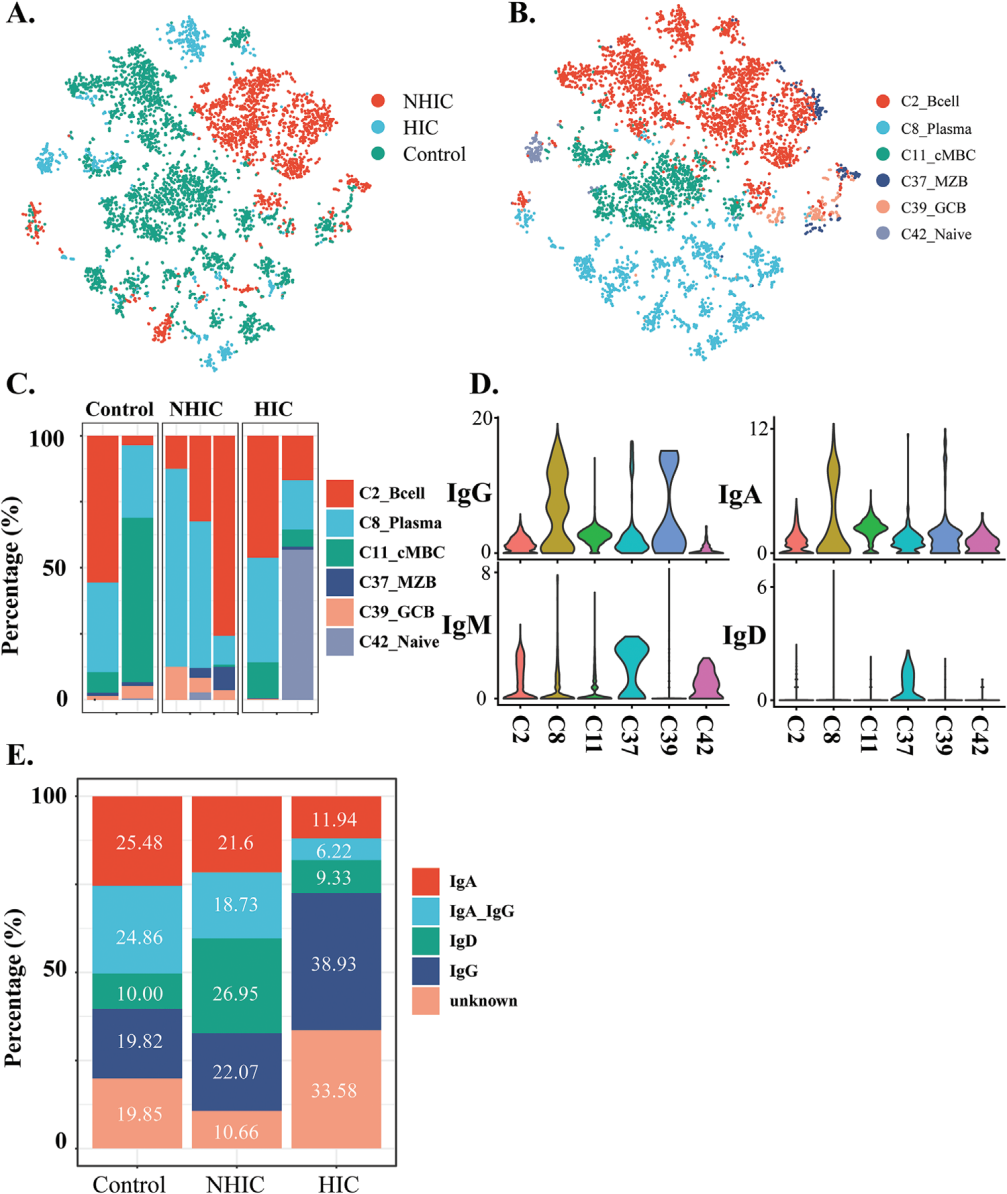
A) *t*‐SNE plot showing B cells from different conditions. B) *t*‐SNE plot showing 6 clusters of 6080 B cells (indicated by colors). C) Cell type frequencies derived from scRNA‐seq of B cell populations shown. D) Heatmap of the AUC scores of TF motifs estimated per cell by SCENIC. Shown are top five differentially activated motifs across different conditions. E) scRNA‐based quantitation of IGHM, IGHD, IGHG, and IGHA expression. IGHG and IGHA values are the sum of subclass counts. Activated cluster contains only naïve activated cells.

B cells clonal expansion has been frequently observed in HIC but not in NHIC. In our study, B cells were subclustered into six distinct subpopulations (**Figure** **5**A,B and Figure S10A, Supporting Information): C2, which expressed *IGHM* and identified IgM^+^ B cells; C8, which expressed *SDC1* and identified the plasma cells, and percentage of it significantly increased in the HIC/NHIC subjects (Figure 5D); C11 highly expressed *KLF2*, which mediates *CD62L* expression and immune cell transendothelial migration into the colonic mucosae, and identified the classical memory B (cMBC) cells.^[^ ^23^ ^]^ C37, which identified naive B cells enriched in the NHIC with strongly expressed *IGHD*, which was absent from HIC and represented a major B‐cell subset in NHIC; C39, which identifies germinal center B (GCB) cells with expressed *BCL‐6* and *MKI67* and C42, which was an IgM^+^IgD^−^ B‐cell subset without CD27 expression and assigned as double negative B cell. Plasma significantly expanded in the NHIC/HIC groups compared to UC (Figure 5C). In contrast with UC, in which IgA was the main immunoglobulin, patients in the NHIC/HIC groups had high concentrations of IgG from B cells (Figure 5E). Heavy chain gene expression in plasma cell cluster was dominated by IGHG, although some cells expressed IGHA genes. Transcriptionally defined memory B cells were observed to express immunoglobulins with a diverse array of isotypes, including *IGHA1*, *IGHG1*, *IGHG3*, *IGHG4*, *IGHM*, and to a lesser extent *IGHD*. The ratio of IgG1 to IgA1 plasma cells was significantly increased in the NHIC/HIC samples. With GSVA, we tested MSigDB immunologic gene sets revealed and GO analyses that IgM^+^, plasma, and MZB groups had relatively high expressions of genes defining the antibody class switching (Figure S10B,C, Supporting Information).

### 2.6 NHIC/HIC Was Characterized by Specific Signaling Networks

**Figure 6:**
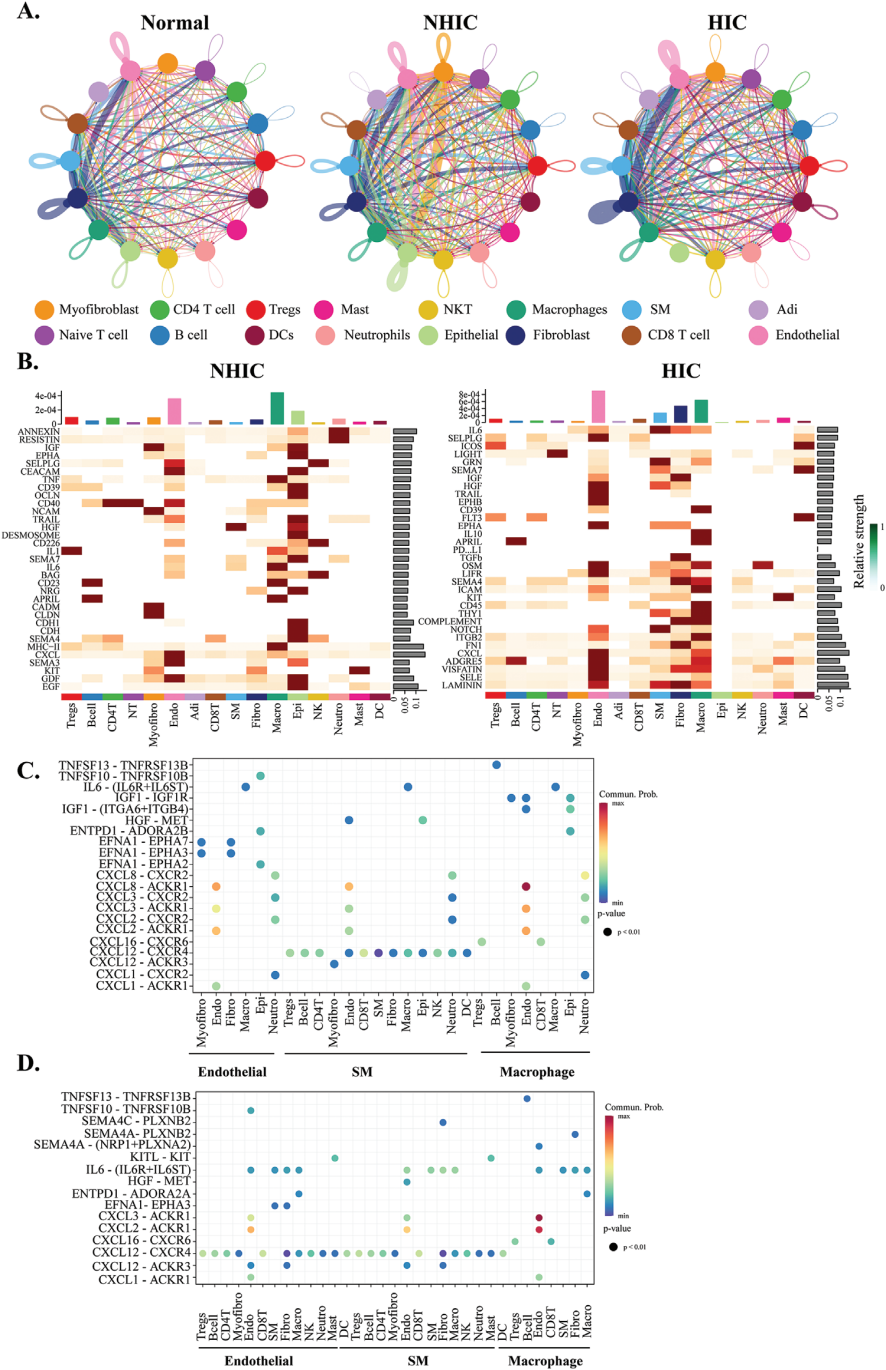
IC/BPS is characterized by cell‐to‐cell signaling networks. A) Chord diagrams of cellular interactome between epithelial, endothelial, fibroblast, and immune cells from different conditions. Networks depicting cell types as nodes and interactions as edges. Size of cell type is proportional to the total number of interactions of each cell type, and edge thickness is proportional to the number of interactions between the connecting types. B) Heatmap of overall signaling among all cell populations compared to the UC. Red (or blue) colored edges represent increased (or decreased) signaling in the second dataset compared to normal samples. Dot plot depicting selected endothelial, fibroblast and macrophages interactions enriched in the C) NHIC and D) HIC compared to the UC.

We used receptor/ligand database and statistical inference framework CellChat to identify potential cell–cell interactions from different groups (**Figure** **6**A,B). In the UC, the cell–cell interaction landscape of the bladder was dominated by structural cells (endothelial cells, epithelial cells, and fibroblasts) communicating with each other by extensive growth factor signaling. In the Figure 6B, the communication relationships between macrophage and endothelial cells were significantly enriched in the NHIC/HIC (*p* < 0.05). The shift in cellular phenotypes in tissue may be due to the local production of pro‐inflammatory cytokines in our NHIC/HIC cohorts. CellChat detected 9503 ligand–receptor pairs among all cell clusters from each group, which were further categorized into 102 signaling pathways (Table S6 and Figure S11A, Supporting Information). Comparison of the information flow for each signaling pathway between NHIC/HIC and UC revealed that 33 signaling pathways were highly active in HIC, mainly involved in immune response, such as CXCL, CSF, IL6, and TNF (Figure S11B, Supporting Information). There were also 12 overlap signaling pathways, that were highly active in both groups, including CXCL, IL6, CD39, IGF, and HGF (Figure S11C, Supporting Information), suggesting pro‐inflammation signaling pathways might critically contribute to disease progression. Specific to CXCL signaling, CellChat identified that ligand–receptor pair CXCL8‐ACKR1 as the most significant signaling pathway in the NHIC samples (Figure 6C), contributing to the communication from macrophage to endothelial cells. In the HIC samples, ligand–receptor pair CXCL2‐ACKR1 and CXCL3‐ACKR1 were found to act as a major signaling pathways from macrophages to endothelial cells (Figure 6D). Together, our results suggested that the activation of TRMs plays an important role in the recruitment of immune cells to bladder microenvironment.^[^ ^24^ ^]^

## 3 Discussion

Here, we presented the cellular landscape of human bladder microenvironment of IC/BPS patients and unaffected controls at single‐cell resolution. By identifying cell subtypes and altered pathways, highlighting the cellular sources of cellular signals, our analyses confirmed many important previous findings and highlighted key areas for further investigations in the biology of structural cells and immune cells, which will fuel advances in IC/BPS diagnosis and therapy.

Herein, we elucidated gene transcription profiles for populations of T cells, including cytotoxic *CD8* ^+^ T, Th1, and Treg cells. In addition, we also detected Th1 of an activated phenotype, and cells expressing genes associated with a type I IFN response signature, which is required for optimal pathogen clearance but also contribute to tissue damage. By contrast, the immune response was heavily skewed toward Th2 immune responses in the urinary tract infection, which are largely directed at bladder tissue repair. Th1 cells were highly activated by TCR signal pathway, possibly due to exposure to self‐antigen of bladder epithelial cells (BEC) damage. Tregs are central to immune homeostasis, and their phenotypic heterogeneity reflects the diverse environments and target cells that they regulate.^[^ ^25^ ^]^ According to a previous study,^[^ ^15^ ^]^ in the presence of IL6, Tregs lost their suppressive capacity and was converted to unstable Tregs to fighting pathogenic disease. Our data demonstrated that IL6‐stimulated Tregs from NHIC/HIC lost the characteristics of immunosuppression. Failure of such regulatory mechanisms can also lead to a state of chronic inflammation.

Myeloid cells are major innate immune phagocytes that constitute the first line of defense against infections in the bladder.^[^ ^18a^ ^]^ The immune response during bladder cystitis is characterized by granulocyte infiltration in the bladder mucosa. In our study, the majority of M1 macrophages were presented in the NHIC samples, while M2 were expanded in the HIC group, suggesting macrophages are pro‐inflammatory in NHIC but anti‐inflammatory in HIC. Functional analysis also showed macrophages were another major player in antigen presentation. As there is a strong link between TRMs and neutrophils migration, chemokines released by macrophages increases the production and recruitment of neutrophils and inhibits the apoptosis of neutrophils, thereby extending their life span in bladder. In particular, fibroblasts formed a positive feedback to maintain an inflammatory environment through the expression of chemokine ligand. As in other autoimmune diseases, inflammatory TRMs that dominate the bladder mucosal milieu have high expressions of the pro‐inflammatory cytokines including IL‐1*β* and TNF‐α.

Our study also highlighted inflammatory fibroblasts as a potential therapeutic target in NHIC/HIC samples. Inflammatory fibroblasts are a major source of pro‐inflammatory cytokines such as IL6.^[^ ^22b^ ^]^ IL6 has been repeatedly linked to IC/BPS, and blockade of IL6 has been shown to alleviate inflammation in preclinical studies of other autoimmune diseases. However, the source of IL6 was typically thought to derive from innate lymphocytes such as macrophages. In our study, IL6 is secreted by activated inflammatory fibroblasts, which was validated by scRNA‐seq and IMC. IL6 is also a key cytokine involved in B cell activation, which explains why plasma cells dramatically increase in IC/BPS patients. Also in our current study, we observed a significantly expanding plasma cells and detected both IgD^+^ and switched B cell populations, which may contribute to the pathogenesis of IC/BPS. The gene expression of B cell markers suggested a balance between germinal center and plasma cell differentiation within the bladder. This observation may serve as a starting point for identifying candidate mechanisms, diagnostic markers, or therapeutics for IC/BPS.

Finally, our ligand–receptor analysis revealed that interactions between members of the bladder microenvironment were complex and involved many signaling pathways. The comprehensive analysis of the cell–cell interactions in NHIC/HIC identifies dominance of TRMs interacting with structural cells. Several studies have highlighted the interconnection between endothelial cells and macrophages in these groups at the level of growth factor and cytokine signaling.^[^ ^24^ ^]^ In our current study, endothelial cells had significant interactions with TRMs. By analyzing the dominant ligand–receptor pairs that mediate the organizations of bladder microenvironment in NHIC/HIC samples, we found that CXCL8‐ACKR1/CXCL2/3‐ACKR1 were ranked top in mediating interactions among endothelial cells and macrophages. Modulating macrophage and/or endothelial functions by interfering with signaling pathways offers new perspectives for therapeutic strategies. This also strongly supports the possibility that TNF‐α signaling alone cannot fully explain the pathogenic cellular organization we identified in the bladder^[^ ^9^, ^26^ ^]^ in terms of TNF‐α blockade, additional inflammatory macrophage‐derived stimulatory mediators such as IL‐1b and OSM are sufficient to trigger pathogenic stromal activation. More interestingly, the treatment of IC/BPS with phosphodiesterase 4 (PDE4) which can inhibit TNF‐α and IL‐6 simultaneously will be an attractive option in our future study.

In summary, we identified the expression profiles of different cells in bladder tissue from NHIC/HIC patients and confirmed the characteristics of different cell types. This cell atlas provides deep insight into the molecular mechanisms of IC/BPS, signaling receptors, and gene data set and will aid in future studies on the relationships among cell types. It also facilitated discovery of novel biomarker for IC/BPS diagnosis and new treatment.

## 4 Experimental Section

### Cohort Recruitment and Sample Collection

IC/BPS patients were subclassified into two subtypes, HIC (*n* = 2) and NHIC (*n* = 3). In addition, two female patients with non‐muscle invasive bladder cancer (NMIBC) were enrolled as UC. In order to validate the result of scRNA‐seq, additional three patients were enrolled for the IMC analysis. IC/BPS diagnosis and subtyping were performed in accordance with the East Asian clinical guidelines and the ESSIC criteria.^[^ ^1b^ ^]^ The clinical characteristics of IC/BPS were further assessed, including the IPSS (International Prostate Symptom Score), OSS (O'Leary‐Saint Score), OABSS (Overactive Bladder Symptoms Score), MBC (Maximal Bladder Capacity under anesthesia), ICSI (Interstitial Cystitis Symptom Index), and ICPI (Interstitial Cystitis Problem Index) in IC/BPS cases. Additional information about these patients are summarized in Table S1 (Supporting Information). The study was approved by the Institutional Review Board of the institution (2020BJYYEC‐141‐01). Written informed consent was obtained from all subjects before study enrollment.

### Tissue Processing

The samples of either NHIC or HIC were harvested from the significant areas of Hunner lesions or glomerulations through cystoscopy. The unaffected normal samples were obtained from the normal areas in patients diagnosed with NMIBC, as far away from the tumor as possible. There was no tumor invasion confirmed by two independent pathologists. The fresh tissues were dissociated into single cells following the previous studies.^[^ ^12^, ^22^ ^]^

### Droplet‐Based scRNA‐seq with 10X Genomics

Single‐cell suspensions were converted to barcoded scRNA‐seq libraries by using the Chromium Single Cell 3’ Library, Gel Bead & Multiplex Kit, and Chip Kit (10X Genomics), aiming for an estimated 10 000 cells per library. Libraries were sequenced on an Illumina HiSeq X‐Ten, and mapped to the human genome (build Hg38) using CellRanger (version: 3.1.0).

### Single‐Cell Gene Expression Quantification and Determination of the Major Cell Types

To call real cells from empty droplets, the emptyDrops() function from R package DropletUtils was used,^[^ ^27^ ^]^ which assesses whether the RNA content associated with a cell barcode is significantly distinct from the ambient background RNA present within each sample. Cells with *p * < 0.05 (Benjamini‐Hochberg‐corrected) were considered for further analysis. Filtered cells were converted to a Seurat object using the Seurat (version 3.1.1).^[^ ^28^ ^]^ Thus, all cells that had either fewer than 200 UMIs, over 6000, or over 15% UMIs derived from mitochondrial genome were removed. From the remaining 44 490 cells, gene expression matrices were normalized to total cellular read count and mitochondrial read count. To reduce dimensionality of this dataset, the variably expressed genes were normalized by SCTransform, and then summarized using *t*‐SNE dimensionality reduction using the default settings. The Clustree package was used to visualize how clusters changed with increasing resolution; also an appropriate resolution (resolution 1.3) was selected.^[^ ^29^ ^]^ To assess the quality of cell cluster identified in this study ROGUE scoring, an entropy‐based metric for assessing the purity of single cell populations, was used.^[^ ^30^ ^]^ To assign clusters and individual cells to cell types, the SingleR^[^ ^31^ ^]^ and clustifyr^[^ ^13^ ^]^ and default parameters with Immgen as the reference dataset were used. With the integrated data, NHIC/HIC data as a benign reference with inferCNV (https://bioconductor.org/packages/infercnv/) were taken. CNVs were estimated based on two major steps: initial CNV (CNVi) calculation and final CNV (CNVf) estimation.

### Identification of Differentially Expressed Genes and Enrichment Analyses

Based on the normalized data, the FindMarkers function was used to identify differentially expressed genes (DEGs). |FoldChange| > 1.5 and adjusted *p* < 0.05 were used as the cut‐off criteria. Gene Set Variation Analysis (GSVA) was performed to determine whether an a priori defined set of genes showed statistically significant, concordant differences on DEGs^[^ ^32^ ^]^ with MSigDB^[^ ^33^ ^]^ collections, including hallmark, KEGG (C2), GO (C5) and immunologic signature gene sets (C7). Gene Ontology (GO) analysis for bulk sequence was performed by using the R package clusterProfiler.^[^ ^34^ ^]^

### Pseudotime Analysis and Branched Gene Expression Analysis

The Monocle2 was used to reconstruct the divergence of cell lineages/trajectories in the analysis.^[^ ^35^ ^]^ Briefly, first Monocle2 was used to estimate size factors, dispersion, and differential gene expression of the target cells, and then the top 1000 most differentially expressed genes were used to order cells in pseudotime. Then the branch with the cells from control group as the root state of the tree were defined. The BEAM feature of Monocle2 was used to define genes that showed significant divergent expression across each branch point in the pseudotime analysis, using default parameters.

### Scoring of Biological Processes

Individual cells were scored for their expressions of gene signatures representing certain biological functions. The AddModuleScore function in Seurat was used to implement the scoring with default settings. The functional signatures were derived from the GO database. Cytotoxicity‐associated genes (GNLY, GZMB, GZMK, IFNG, and NKG7), co‐stimulation (CD28, CD226, TNFRSF4, TNFRSF9, ICOS, CD27, TNFSF14, CD80, TNFSF4, CD86, TNFSF11, CD276, CD40LG, and TNFRSF18), and six well‐defined exhaustion markers (LAG3, TIGIT, PDCD1, CTLA4, HAVCR2, and TOX) were used to define the cytotoxicity, stimulation, and exhaustion score, respectively. Apoptosis score was measured by the upregulation of the integrated proapoptotic pathways and downregulation of pro‐survival gene expression.

### Macrophage Polarization Analysis

For macrophage polarization analysis, MacSpectrum was used, a single‐cell RNA‐sequencing based gene enrichment tool to infer the macrophage activity of the immune population.^[^ ^36^ ^]^ It estimates the Macrophage Polarization Index (MPI) and Activation induced Macrophage Differentiation Index (AMDI) based on input RNA‐seq count data; both scores have a range from −50 to 50. A higher MPI value indicates greater pro‐inflammatory features and a higher AMDI value indicates greater maturity. In the current analysis, zero was used as a threshold to define “pre‐activation” or “M0” cells (AMDI < 0, MPI < 0), “M1‐transitional” or “M1 pre‐activation” cells (AMDI < 0, MPI > 0), “M2‐like” cells (AMDI > 0, MPI < 0), and “M1‐like” cells (AMDI > 0, MPI > 0).

### Gene Regulated Network Analysis

SCENIC was run as described in the literature.^[^ ^37^ ^]^ In the current study, the motif databases that allow the use of RcisTarget (version: 0.99) were used. The 20 003 motifs (Position Weight Matrices) in the cisTarget database (v8) on the regulatory regions up to 5 KB upstream of each gene and within its introns were scored. The gene‐motif rankings were then built taking the best‐scoring region for each motif and gene.

### Cell to Cell Intercommunication Analysis

To enable a systematic analysis of cell–cell communication molecules, CellChat was used.^[^ ^38^ ^]^ CellChat is a manual curated repository of ligands, receptors, and their interactions, integrated with a new statistical framework for inferring cell–cell communication networks from single cell transcriptome data.

### RNA Velocity Analysis

RNA velocity analysis was performed using the velocyto.R program (http://velocyto.org, version 0.6).^[^ ^39^ ^]^ Briefly, spliced/unspliced reads were annotated by velocyto.py with CellRanger, which generated BAM files and an accompanying GTF; then they were saved in .loom files. After the cells in the bottom 0.5% of the total unspliced transcript count were filtered out, genes were removed according to an average spliced variant expression of 0.2 or an average unspliced variant expression of 0.05 in at least one cluster. Lastly, the velocity vector arrows were projected onto the *t*‐SNE plot which was obtained in Seurat.

### Imaging Mass Cytometry

The antibody panel includes lymphocyte types, cytokine expression, lymphocyte activation, vascular and spatial structure of tissue cells. Descriptions of antibodies, isotope tags, clones and concentrations used for staining can be found in Table S5 (Supporting Information). According to the results of H&E staining, a region of 500 × 500 µm was selected for IMC analysis with a Hyperion Imaging System (Fluidigm) according to manufacturer instructions. The largest square area was laser‐ablated in a rastered pattern at 200 Hz, and preprocessing of the raw data was completed using MCD Viewer (Fluidigm). Data were converted to TIFF format and segmented into single cells using the flexible analysis pipeline (https://github.com/BodenmillerGroup/ImcSegmentationPipeline). In brief, individual cells and regions were segmented using a combination of Ilastik v.1.3^[^ ^40^ ^]^ and CellProfiler v.4.6.^[^ ^41^ ^]^ Ilastik was used to generate a probability map by classifying pixels on the basis of a combination of antibody stains to identify membranes and nuclei. Probability maps were then segmented into single‐cell object masks using CellProfiler. Single‐cell segmentation masks and TIFF images of the 32 channels were overlaid and the mean expression levels of markers and spatial features of single cells were extracted using the histoCAT.

### Correlation to Public Dataset

IC/BPS samples (GSE11783) in the GEO databases were downloaded and normalized.^[^ ^16^ ^]^ DEGs analysis was performed with Limma^[^ ^42^ ^]^ to evaluate the relative abundance of each cell type identified in the present study. Gene signatures from scRNA‐seq were evaluated with GSVA.

### Statistical Analyses

For the scRNA‐seq data, statistical analyses and graphics production were performed using R (v3.6.3) and python. In general, non‐parametric tests were used and *p* value after false discovery correction procedures < 0.05 were considered as statistically significant. For the experimental data, graphics were produced using ggplot2. Detailed descriptions of statistical tests are specified in Section 2 and in the figure legends.

## Conflict of Interest

The authors declare no conflict of interest.

## Supporting information

Supporting Information

Supporting Information

## Data Availability

The data that support the findings of this study are openly available in Genome Sequence Archive for Human (GSA‐Human) at https://ngdc.cncb.ac.cn/, reference number 4966.

## References

[1] a) Y. Homma , Y. Akiyama , H. Tomoe , A. Furuta , T. Ueda , D. Maeda , A. T. Lin , H. C. Kuo , M. H. Lee , S. J. Oh , J. C. Kim , K. S. Lee , Int. J. Urol. 2020, 27, 578; 32291805 10.1111/iju.14234

[2] Y. Akiyama , D. Maeda , H. Katoh , T. Morikawa , A. Niimi , A. Nomiya , Y. Sato , T. Kawai , A. Goto , T. Fujimura , H. Fukuhara , T. Nakagawa , Y. Igawa , S. Ishikawa , M. Fukayama , H. Kume , Y. Homma , J. Urol. 2019, 202, 290. 30865573 10.1097/JU.0000000000000234

[3] J. C. Nickel , R. C. Doiron , Eur. Urol. 2020, 78, e122. 32507337 10.1016/j.eururo.2020.04.067

[4] M. Gamper , V. Viereck , J. Eberhard , J. Binder , C. Moll , J. Welter , R. Moser , Int. Urogynecol. J. 2013, 24, 2049. 23670165 10.1007/s00192-013-2112-0 PMC3838592

[5] M. M. Jensen , W. Jia , A. J. Schults , X. Ye , G. D. Prestwich , S. Oottamasathien , Cytokine 2018, 110, 420. 29784508 10.1016/j.cyto.2018.05.012 PMC6103803

[6] Y. H. Jiang , J. F. Jhang , Y. H. Hsu , H. C. Ho , Y. H. Wu , H. C. Kuo , Sci. Rep. 2021, 11, 914. 33441752 10.1038/s41598-020-80131-5 PMC7806856

[7] T. Yamada , Int. J. Urol. 2003, 10, 463,. 12941123 10.1046/j.1442-2042.2003.00664.x

[8] J. F. Jhang , Y. H. Hsu , C. W. Peng , Y. H. Jiang , H. C. Ho , H. C. Kuo , J. Urol. 2018, 200, 590. 29653163 10.1016/j.juro.2018.03.133

[9] P. C. Bosch , Eur. Urol. 2018, 74, 623. 30072210 10.1016/j.eururo.2018.07.026

[10] a) F. Zhang , K. Wei , K. Slowikowski , C. Y. Fonseka , D. A. Rao , S. Kelly , S. M. Goodman , D. Tabechian , L. B. Hughes , K. Salomon‐Escoto , G. F. M. Watts , A. H. Jonsson , J. Rangel‐Moreno , N. Meednu , C. Rozo , W. Apruzzese , T. M. Eisenhaure , D. J. Lieb , D. L. Boyle , A. M. Mandelin, 2nd , Accelerating Medicines Partnership Rheumatoid Arthritis and Systemic Lupus Erythematosus Consortium , B. F. Boyce , E. DiCarlo , E. M. Gravallese , P. K. Gregersen , L. Moreland , G. S. Firestein , N. Hacohen , C. Nusbaum , et al., Nat. Immunol. 2019, 20, 928;31061532

[11] P. Zhang , M. Yang , Y. Zhang , S. Xiao , X. Lai , A. Tan , S. Du , S. Li , Cell Rep. 2019, 27, 1934. 31067475 10.1016/j.celrep.2019.04.052

[12] Z. Yu , J. Liao , Y. Chen , C. Zou , H. Zhang , J. Cheng , D. Liu , T. Li , Q. Zhang , J. Li , X. Yang , Y. Ye , Z. Huang , X. Long , R. Yang , Z. Mo , J. Am. Soc. Nephrol. 2019, 30, 2159. 31462402 10.1681/ASN.2019040335 PMC6830796

[13] R. Fu , A. E. Gillen , R. M. Sheridan , C. Tian , M. Daya , Y. Hao , J. R. Hesselberth , K. A. Riemondy , F1000Research 2020, 9, 223. 32765839 10.12688/f1000research.22969.1 PMC7383722

[14] J. Wu , C. Bao , R. L. Reinhardt , S. N. Abraham , Proc. Natl. Acad. Sci. U. S. A. 2021, 118, e2026461118. 33653961 10.1073/pnas.2026461118 PMC7958421

[15] Y. Z. Gang Yi , F. Xie , F. Zhu , Z. Wan , J. Wang , X. Wang , K. Gao , L. Cao , X. Li , C. Chen , Y. Kuang , X. Qiu , H. Yang , J. Wang , B. Su , L. Chen , W. Zhang , Y. Hou , X. Xu , Y. He , A. Tsun , X. Liu , B. Li , Sci. Bull. 2020, 65, 1114. 10.1016/j.scib.2020.01.002 36659163

[16] M. Gamper , V. Viereck , V. Geissbuhler , J. Eberhard , J. Binder , C. Moll , H. Rehrauer , R. Moser , BMC Genomics 2009, 10, 199. 19400928 10.1186/1471-2164-10-199 PMC2686735

[17] I. M. Djuretic , D. Levanon , V. Negreanu , Y. Groner , A. Rao , K. M. Ansel , Nat. Immunol. 2007, 8, 145. 17195845 10.1038/ni1424

[18] a) L. Lacerda Mariano , M. A. Ingersoll , Cell. Immunol. 2018, 330, 136; 29422271 10.1016/j.cellimm.2018.01.018

[19] A. Mozaffarian , A. W. Brewer , E. S. Trueblood , I. G. Luzina , N. W. Todd , S. P. Atamas , H. A. Arnett , J. Immunol. 2008, 181, 7243. 18981146 10.4049/jimmunol.181.10.7243

[20] S. P. Short , J. M. Pilat , C. W. Barrett , V. K. Reddy , Y. Haberman , J. R. Hendren , B. J. Marsh , C. E. Keating , A. K. Motley , K. E. Hill , A. E. Zemper , M. K. Washington , C. Shi , X. Chen , K. T. Wilson , J. S. Hyams , L. A. Denson , R. F. Burk , M. J. Rosen , C. S. Williams , Gastroenterology 2021, 160, 1694. 33388316 10.1053/j.gastro.2020.12.059 PMC8035252

[21] S. Alivernini , L. MacDonald , A. Elmesmari , S. Finlay , B. Tolusso , M. R. Gigante , L. Petricca , C. Di Mario , L. Bui , S. Perniola , M. Attar , M. Gessi , A. L. Fedele , S. Chilaka , D. Somma , S. N. Sansom , A. Filer , C. McSharry , N. L. Millar , K. Kirschner , A. Nerviani , M. J. Lewis , C. Pitzalis , A. R. Clark , G. Ferraccioli , I. Udalova , C. D. Buckley , E. Gremese , I. B. McInnes , T. D. Otto , et al., Nat. Med. 2020, 26, 1295. 32601335 10.1038/s41591-020-0939-8

[22] a) M. Bartoschek , N. Oskolkov , M. Bocci , J. Lovrot , C. Larsson , M. Sommarin , C. D. Madsen , D. Lindgren , G. Pekar , G. Karlsson , M. Ringner , J. Bergh , A. Bjorklund , K. Pietras , Nat. Commun. 2018, 9, 5150; 30514914 10.1038/s41467-018-07582-3 PMC6279758

[23] D. Bhattacharya , M. T. Cheah , C. B. Franco , N. Hosen , C. L. Pin , W. C. Sha , I. L. Weissman , J. Immunol. 2007, 179, 6808. 17982071 10.4049/jimmunol.179.10.6808 PMC4517294

[24] T. Girbl , T. Lenn , L. Perez , L. Rolas , A. Barkaway , A. Thiriot , C. Del Fresno , E. Lynam , E. Hub , M. Thelen , G. Graham , R. Alon , D. Sancho , U. H. von Andrian , M. B. Voisin , A. Rot , S. Nourshargh , Immunity 2018, 49, 1062. 30446388 10.1016/j.immuni.2018.09.018 PMC6303217

[25] C. Dejaco , C. Duftner , B. Grubeck‐Loebenstein , M. Schirmer , Immunology 2006, 117, 289. 16476048 10.1111/j.1365-2567.2005.02317.x PMC1782226

[26] P. C. Bosch , J. Urol. 2014, 191, 77. 23792149 10.1016/j.juro.2013.06.038

[27] A. T. L. Lun , S. Riesenfeld , T. Andrews , T. P. Dao , T. Gomes , J. participants in , the 1st Human Cell Atlas , J. C. Marioni , Genome Biol. 2019, 20, 63. 30902100 10.1186/s13059-019-1662-y PMC6431044

[28] T. Stuart , A. Butler , P. Hoffman , C. Hafemeister , E. Papalexi , W. M. Mauck, 3rd , Y. Hao , M. Stoeckius , P. Smibert , R. Satija , Cell 2019, 177, 1888. 31178118 10.1016/j.cell.2019.05.031 PMC6687398

[29] L. Zappia , A. Oshlack , Gigascience 2018, 7, 083. 10.1093/gigascience/giy083 PMC6057528 30010766

[30] B. Liu , C. Li , Z. Li , D. Wang , X. Ren , Z. Zhang , Nat. Commun. 2020, 11, 3155. 32572028 10.1038/s41467-020-16904-3 PMC7308400

[31] H. Li , E. T. Courtois , D. Sengupta , Y. Tan , K. H. Chen , J. J. L. Goh , S. L. Kong , C. Chua , L. K. Hon , W. S. Tan , M. Wong , P. J. Choi , L. J. K. Wee , A. M. Hillmer , I. B. Tan , P. Robson , S. Prabhakar , Nat. Genet. 2017, 49, 708. 28319088 10.1038/ng.3818

[32] S. Hanzelmann , R. Castelo , J. Guinney , BMC Bioinformatics 2013, 14, 7. 23323831 10.1186/1471-2105-14-7 PMC3618321

[33] A. Liberzon , C. Birger , H. Thorvaldsdottir , M. Ghandi , J. P. Mesirov , P. Tamayo , Cell Syst. 2015, 1, 417. 26771021 10.1016/j.cels.2015.12.004 PMC4707969

[34] G. Yu , L. G. Wang , Y. Han , Q. Y. He , OMICS 2012, 16, 284. 22455463 10.1089/omi.2011.0118 PMC3339379

[35] X. Qiu , A. Hill , J. Packer , D. Lin , Y. A. Ma , C. Trapnell , Nat. Methods 2017, 14, 309. 28114287 10.1038/nmeth.4150 PMC5330805

[36] C. Li , A. Menoret , C. Farragher , Z. Ouyang , C. Bonin , P. Holvoet , A. T. Vella , B. Zhou , JCI Insight 2019, 4, e126453. 10.1172/jci.insight.126453 PMC6542613 30990466

[37] S. Aibar , C. B. Gonzalez‐Blas , T. Moerman , V. A. Huynh‐Thu , H. Imrichova , G. Hulselmans , F. Rambow , J. C. Marine , P. Geurts , J. Aerts , J. van den Oord , Z. K. Atak , J. Wouters , S. Aerts , Nat. Methods 2017, 14, 1083. 28991892 10.1038/nmeth.4463 PMC5937676

[38] R. Vento‐Tormo , M. Efremova , R. A. Botting , M. Y. Turco , M. Vento‐Tormo , K. B. Meyer , J. E. Park , E. Stephenson , K. Polanski , A. Goncalves , L. Gardner , S. Holmqvist , J. Henriksson , A. Zou , A. M. Sharkey , B. Millar , B. Innes , L. Wood , A. Wilbrey‐Clark , R. P. Payne , M. A. Ivarsson , S. Lisgo , A. Filby , D. H. Rowitch , J. N. Bulmer , G. J. Wright , M. J. T. Stubbington , M. Haniffa , A. Moffett , S. A. Teichmann , Nature 2018, 563, 347. 30429548 10.1038/s41586-018-0698-6 PMC7612850

[39] G. L. Manno , R. Soldatov , A. Zeisel , E. Braun , H. Hochgerner , V. Petukhov , K. Lidschreiber , M. E. Kastriti , P. Lonnerberg , A. Furlan , J. Fan , L. E. Borm , Z. Liu , D. van Bruggen , J. Guo , X. He , R. Barker , E. Sundstrom , G. Castelo‐Branco , P. Cramer , I. Adameyko , S. Linnarsson , P. V. Kharchenko , Nature 2018, 560, 494. 30089906 10.1038/s41586-018-0414-6 PMC6130801

[40] S. Berg , D. Kutra , T. Kroeger , C. N. Straehle , B. X. Kausler , C. Haubold , M. Schiegg , J. Ales , T. Beier , M. Rudy , K. Eren , J. I. Cervantes , B. Xu , F. Beuttenmueller , A. Wolny , C. Zhang , U. Koethe , F. A. Hamprecht , A. Kreshuk , Nat. Methods 2019, 16, 1226. 31570887 10.1038/s41592-019-0582-9

[41] C. McQuin , A. Goodman , V. Chernyshev , L. Kamentsky , B. A. Cimini , K. W. Karhohs , M. Doan , L. Ding , S. M. Rafelski , D. Thirstrup , W. Wiegraebe , S. Singh , T. Becker , J. C. Caicedo , A. E. Carpenter , PLoS Biol. 2018, 16, e2005970. 29969450 10.1371/journal.pbio.2005970 PMC6029841

[42] M. E. Ritchie , B. Phipson , D. Wu , Y. Hu , C. W. Law , W. Shi , G. K. Smyth , Nucleic Acids Res. 2015, 43, e47. 25605792 10.1093/nar/gkv007 PMC4402510

