# Supplementary material for: Multimodal Single‐Cell Analyses Outline the Immune Microenvironment and Therapeutic Effectors of Interstitial Cystitis/Bladder Pain Syndrome
Source: Adv Sci (Weinh). 2022 Apr 25;9(18):2106063. doi: 10.1002/advs.202106063 (PMC9218658; doi:10.1002/advs.202106063)
Supplement: Supplementary file 1 — Supporting Information [file ADVS-9-2106063-s002.pdf]

ADVANCED SCIENCE
Open Access

Supporting Information

for *Adv. Sci.*, DOI 10.1002/advs.202106063

Multimodal Single-Cell Analyses Outline the Immune Microenvironment and Therapeutic Effectors of Interstitial Cystitis/Bladder Pain Syndrome

Fei Su, Wei Zhang, Lingfeng Meng, Wei Zhang, Xiaodong Liu, Xiaorui Liu, Meng Chen, Yaoguang Zhang and Fei Xiao**

Title:

Multimodal single-cell analyses reveal the landscape of bladder microenvironment in interstitial cystitis/bladder pain syndrome

Fei Su[1,4], Wei Zhang[2], Lingfeng Meng[3], Wei Zhang[3], Xiaodong Liu[3], Xiaorui Liu[5], Meng Chen[6], Yaoguang Zhang[3†], Fei Xiao[1,4†]

[1]Clinical Biobank, [2]Department of Pathology, [3]Department of Urology, [4]The Key Laboratory of Geriatrics, Beijing Hospital, National Center of Gerontology, Institute of Geriatric Medicine, Chinese Academy of Medical Sciences, Beijing 100730, P.R. China.

[5]Shanghai Key Laboratory of Embryo Original Diseases, The International Peace Maternity and Child Health Hospital, School of Medicine, Shanghai Jiao Tong University, Shanghai 200030, P.R. China.

[6] Key Laboratory for National Cancer Big Data Analysis and Implement, National Cancer Data Center, National Cancer Center/National Clinical Research Center for Cancer/Cancer Hospital, Chinese Academy of Medical Sciences and Peking Union Medical College, Beijing, 100021, P.R. China

[†]**Correspondence to:**

Yaoguang Zhang (zhangyaoguang3247@bjhmoh.cn)

Fei Xiao (xiaofei3965@bjhmoh.cn)

Supplementary Figures

Figure S1 Bioinformatics approach to identification and removal of stressed or tumor cells.

Figure S2 Quality assessment of cell cluster and annotation

Figure S3 Functional analysis of differentially expressed genes from different T/NK cells

Figure S4 Velocity field projected onto *t*-SNE plot of the cell states of $CD4^+$ T cells

Figure S5 Boxplot of cytotoxicity, PMN, co-stimulatory, co-inhibtory, inflammation and IgG scores defined by related genes for the three conditions

Figure S6 Hematoxylin and eosin (H&E) staining of the samples for imaging mass cytometry

Figure S7 Functional analysis of differentially expressed genes from different myeloid cell types

Figure S8 Distribution of activated macrophage cells predicted by MacSpectrum in the different conditions

Figure S9 Gene expression analysis of state-specific genes from Monocle2

Figure S10 Functional analysis of differentially expressed genes from different B cells

Figure S11 Analysis of signaling pathways involved in the cell-cell interaction.

Figure S1

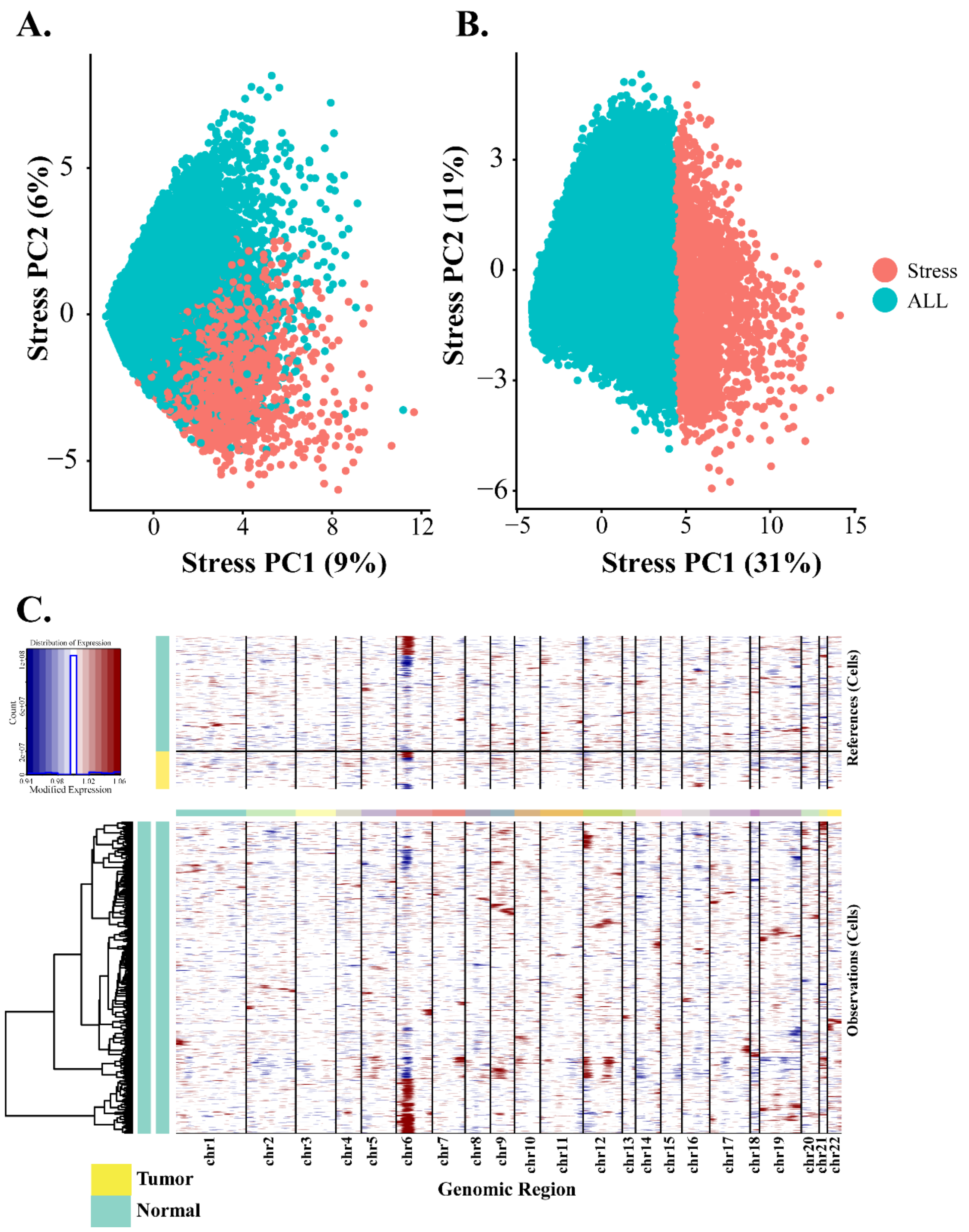

Figure S1:

Bioinformatics approach to identification and removal of stressed and tumor cells.

A. The signature of cell stress from an MSigDB list of stress response genes (M10970) was used to create a pseudogene for principal component analysis

B. The signature of cell stress from stress gene signature was used to create a pseudogene for principal component analysis

C. Large-scale CNVs of single cells (rows). CNVs were inferred from transcriptomes. Red, amplifications; blue, deletions.

Figure S2

A.

B.

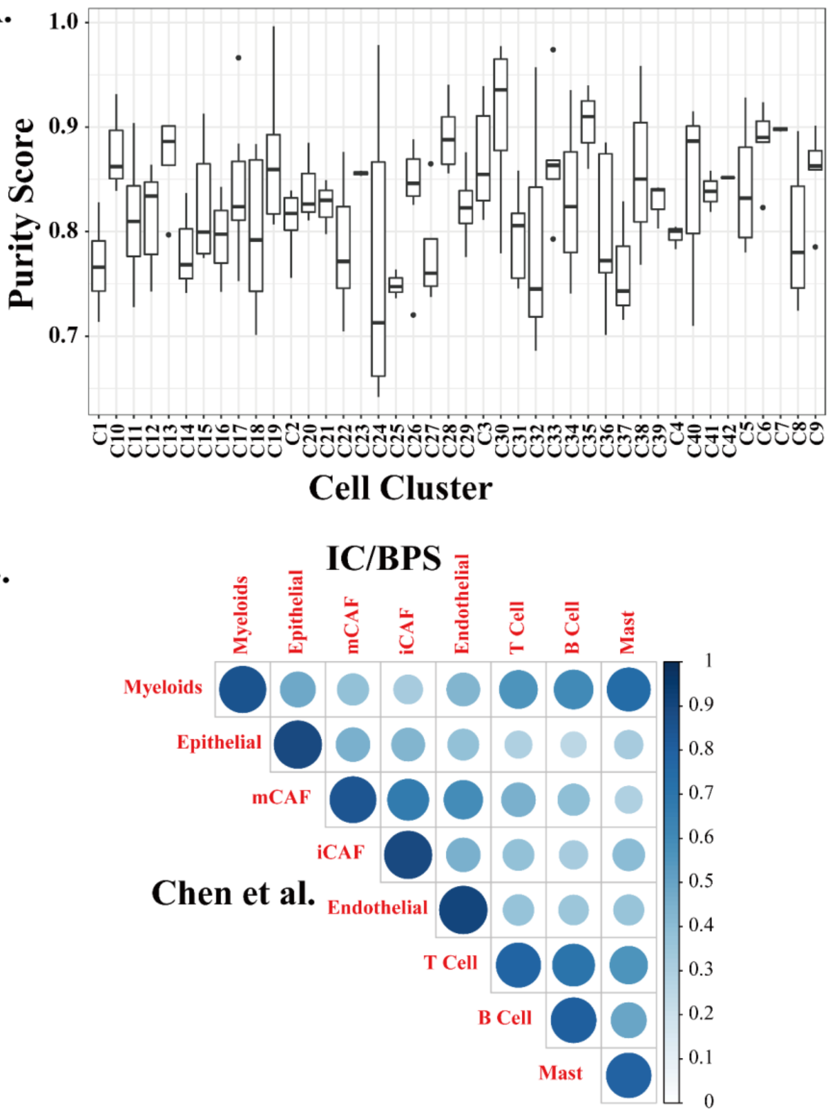

Figure S2:

Quality assessment of cell cluster and annotation

A. Cluster quality score determined by ROGUE analysis. The x-axis stands for different cell clusters and y-axis stands for the purity score from ROGUE.

B. Spearman correlation scores between cell clusters from this study and cell clusters from bladder cancer (Chen et al. doi: 10.1038/s41467-020-18916-5)

Figure S3

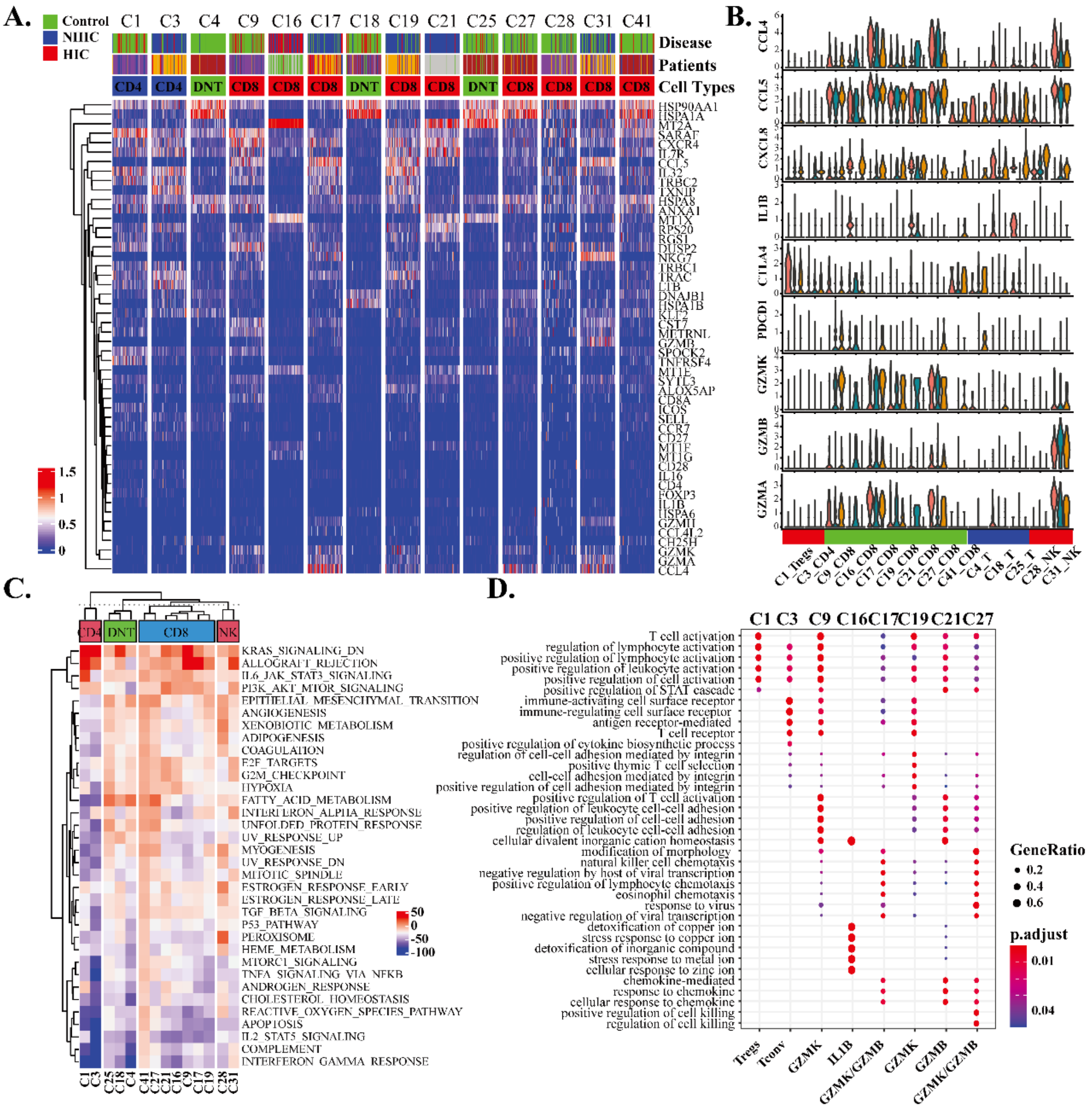

Figure S3:

Functional analysis of differentially expressed genes from different T/NK cells

A. Heatmap of differentially expressed genes (DEGs) of every cell types from different cell clusters. The top 10 DEGs were selected using the Wilcoxon rank-sum test, and ranked based on their read counts. In the top panels, rows correspond to disease groups, patients and cell-types.

B. Violin plot show expression level of key cytokines and chemokines highly expressed in T cells. Different colors stand for disease groups: red for unaffected

control; green for NHIC; orange for HIC.

C. Heatmap shows difference in pathway activities of hallmark pathways scored by GSVA among different clusters of T/NK cells. Shown are *t*-values from a lineal model. Top boxes shows the cell lineages with different colors.

D. Enriched Gene Ontology (GO) biological processes of upregulated genes in different clusters, with gene ratio and adjusted p-value. Enrichment ratio was computed as the ratio of the observed gene count to background gene count. Significance was determined by adjusted *p*-value less than 0.05.

Figure S4

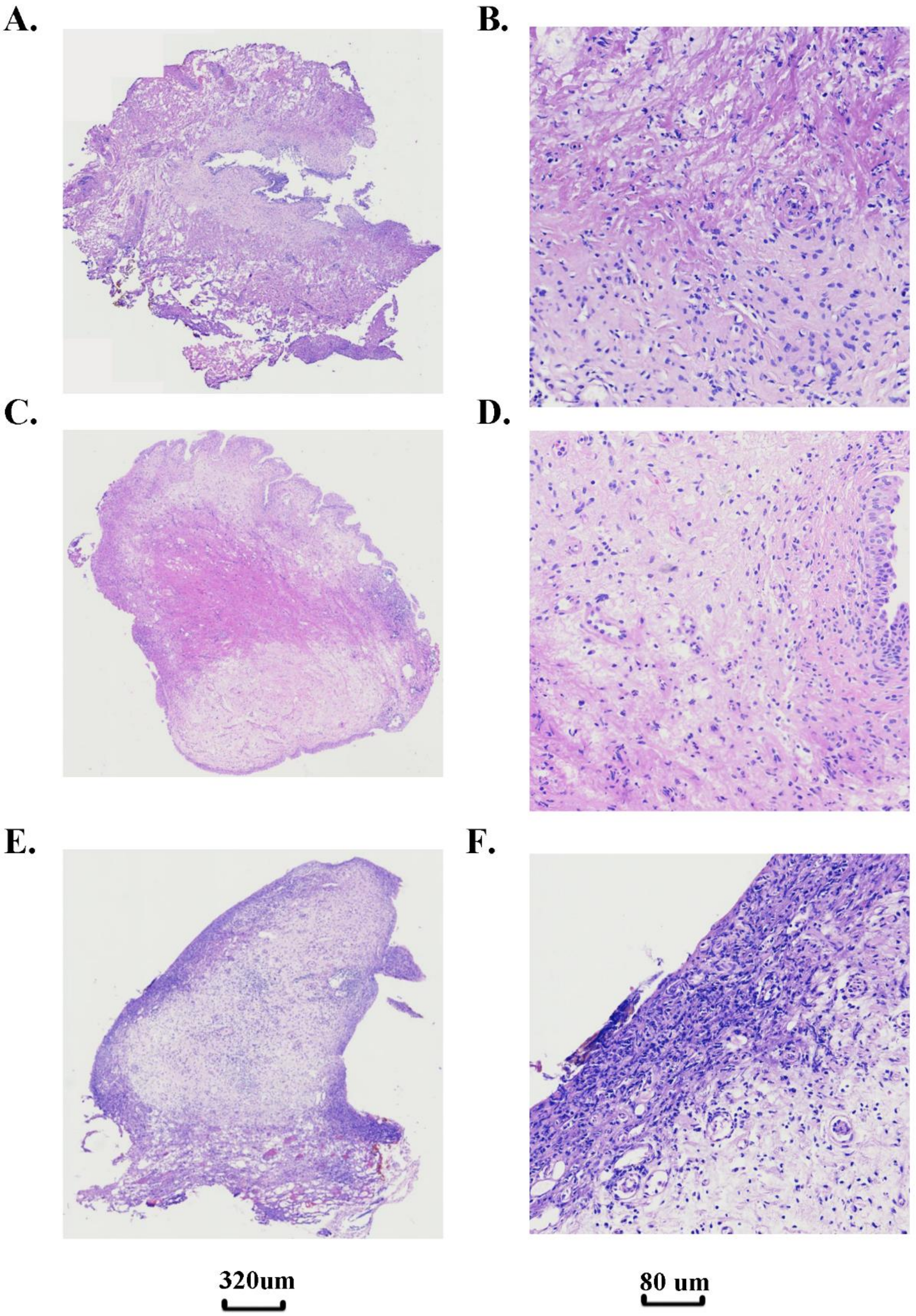

Figure S4:

Hematoxylin and eosin (H&E) staining of the samples for imaging mass cytometry. Whole slide (A; 20x) and selected section (B; 100x) are the same slide from

unaffected normal sample. C (20x) and D (100x) are from NHIC. E (20x) and F (100x) are from HIC. The scale bars of the same column are on the bottom.

Figure S5

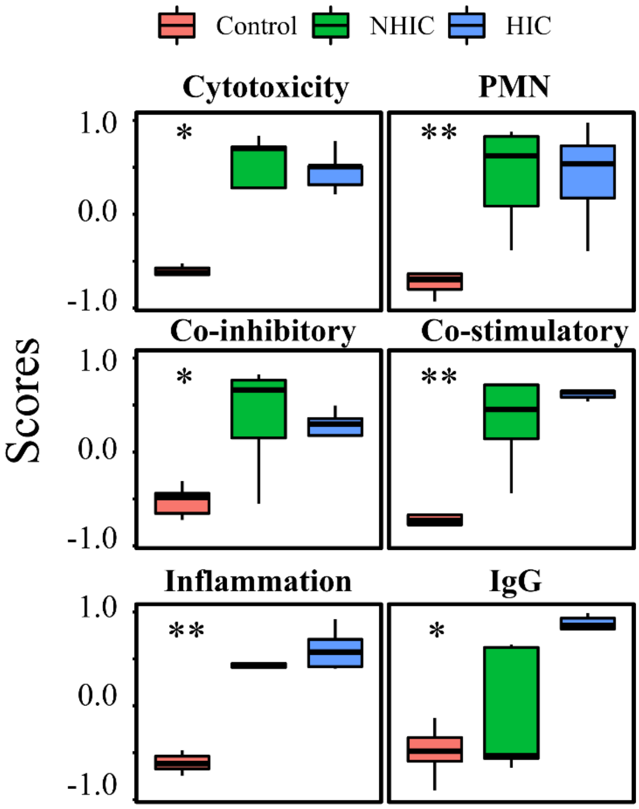

Figure S5:

Boxplot of cytotoxicity, PMN, co-stimulatory, co-inhibtory, inflammation and IgG scores defined by related genes for the three conditions: unaffected control (red), NHIC (green) and HIC (blue). Significance was determined by Student's *t*-test. NS, not significant ($P > 0.05$); * stands for $P \le 0.05$; ** stands for $P \le 0.05$; *** stands for $P \le 0.05$; **** stands for $P \le 0.05$;

Figure S6

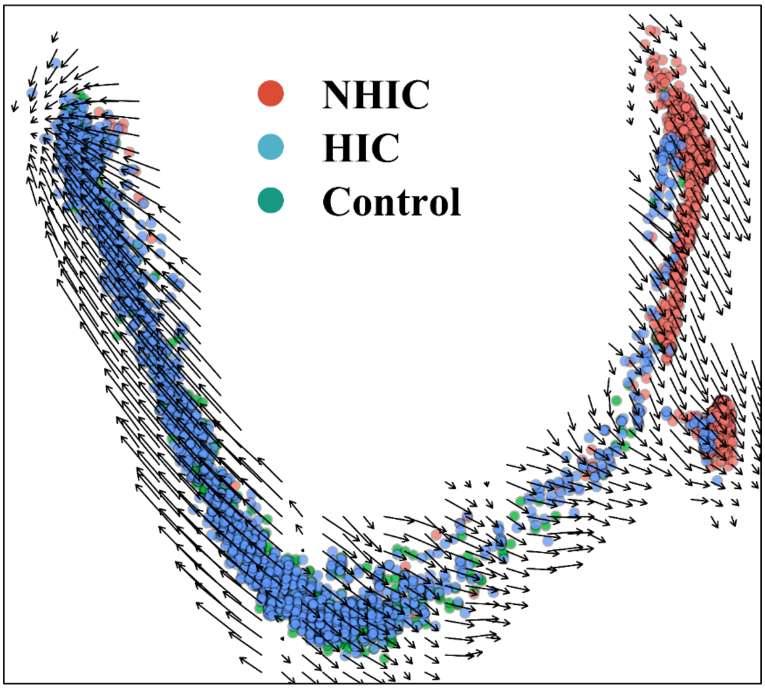

Figure S6:

Velocity field projected onto *t*-SNE plot of the cell states of CD4^{+} T cells

Figure S7

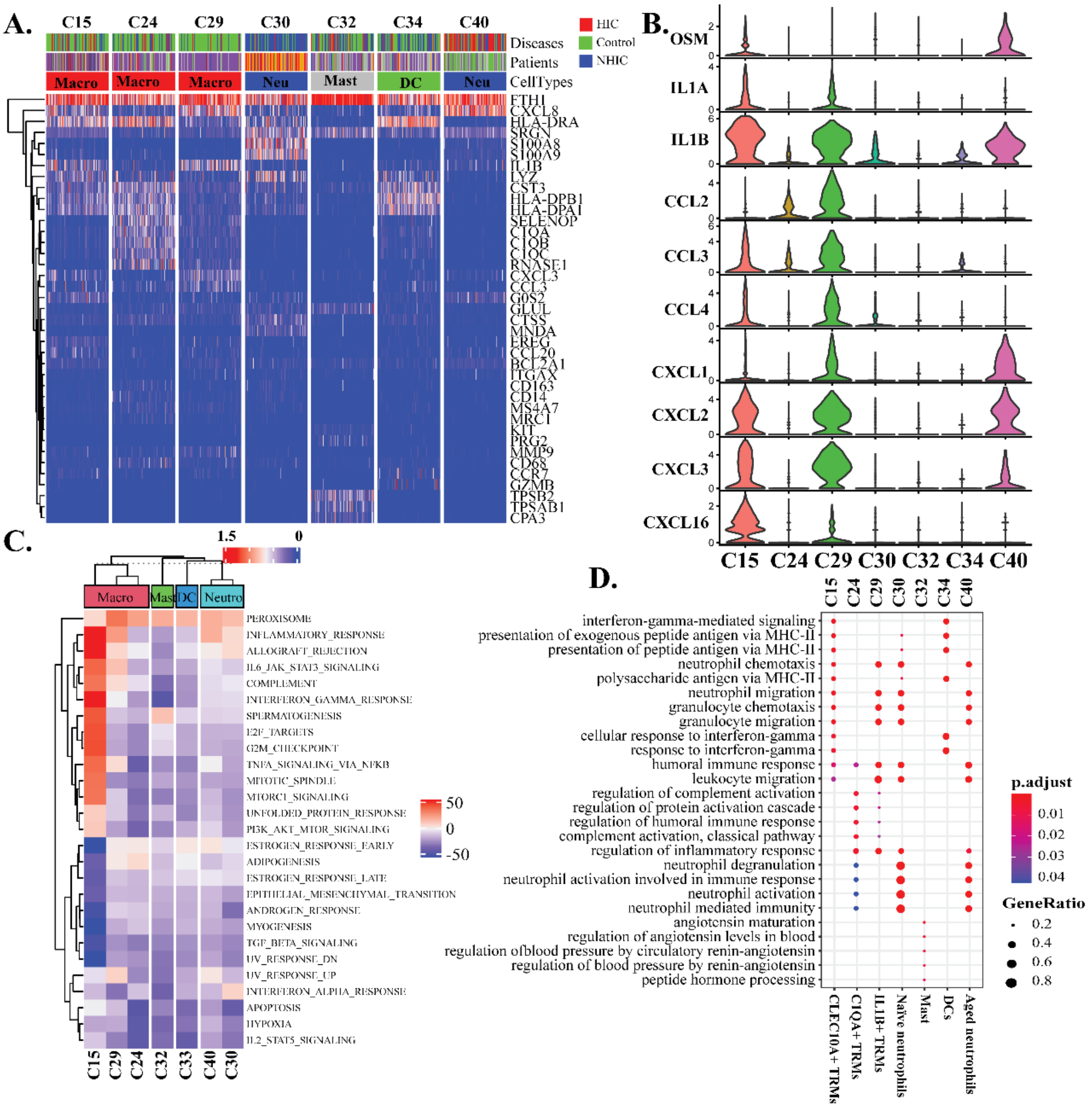

Figure S7:

Functional analysis of differentially expressed genes from different myeloid cell types

A. Heatmap of differentially expressed genes (DEGs) of every cell types from different clusters of myeloid cells.

B. Violin plot show expression level of key cytokines and chemokines highly expressed in myeloid cells

C. Heatmap shows difference in pathway activities of hallmark pathways scored by GSVA among different clusters of myeloid cells. Shown are *t*-values from a lineal

model. Top boxes shows the cell lineages with different colors.

D. Enriched Gene Ontology (GO) biological processes of upregulated genes in different clusters. Enrichment ratio was computed as the ratio of the observed gene count to background gene count. Significance was determined by adjusted *p*-value less than 0.05.

Figure S8

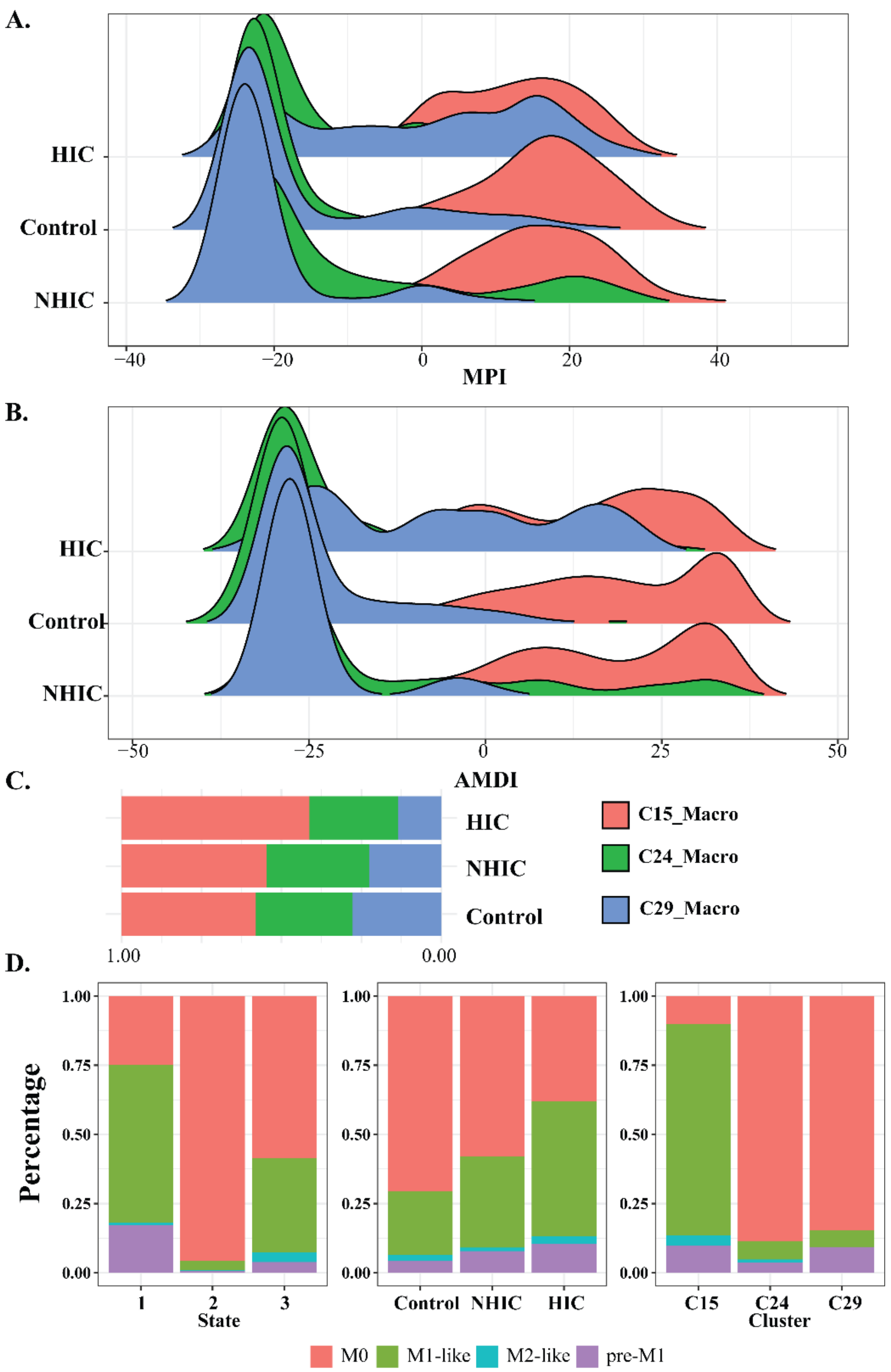

Figure S8:

Distribution of activated macrophage cells predicted by MacSpectrum in the different conditions

MPI (A) and AMDI (B) density curve for all macrophage cells in the different

conditions.

C. Percentage of different cell clusters in the conditions. Different colors stand for distinct cell clusters: red (C15), green (C24) and blue (C29).

D. Percentage of different types of macrophages predicted by MacSpectrum in the cell states (A), conditions (B) and cell clusters (C). Different colors stand for distinct macrophage types: M0 (red), M1-like (green), M2-like (blue) and pre-M1 (purple).

Figure S9

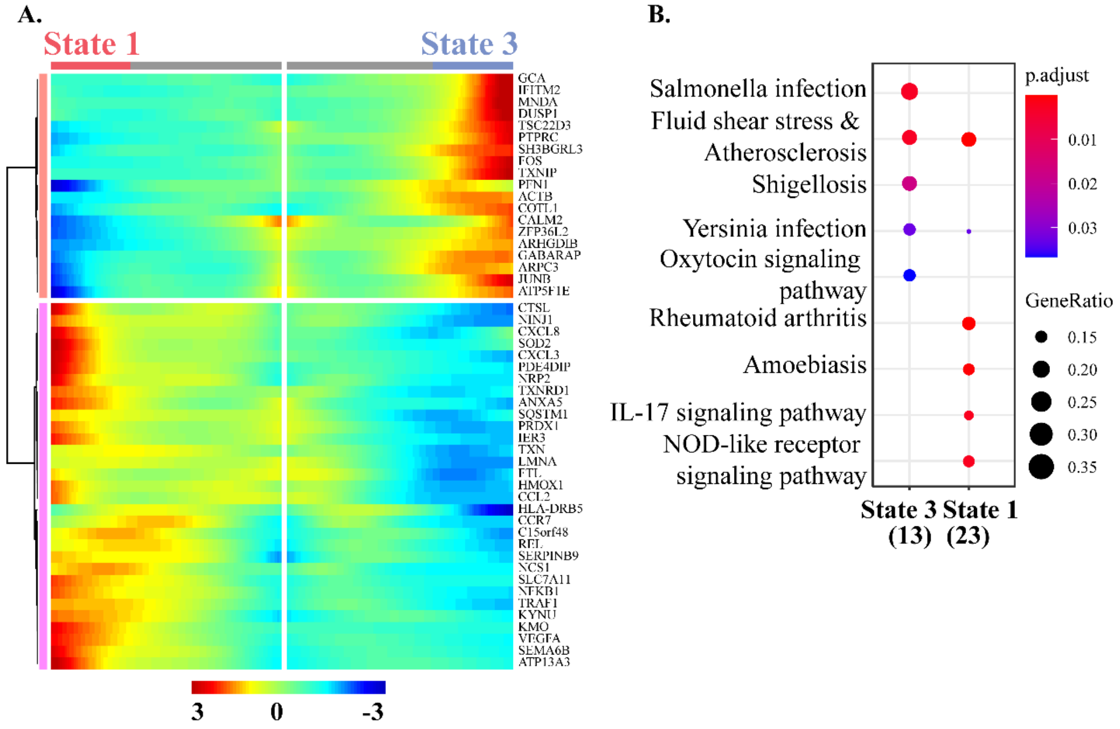

Figure S9:

Gene expression analysis of state-specific genes from Monocle2

A. Enriched GO functions of upregulated genes in different cell states of Monocle2. Top five enriched pathway calculated by Monocle2 were shown as dot plots displayed as expression level over pseudotime.

B. GO analysis of DEGs in distinct cell states using the clusterProfiler. Selected terms are represented in a dot plot, with the size of the dot corresponding to the number of genes per term and the color of the dots corresponding to the adjust p-value of enrichment after $-\log_{10}$ transformation. Differentially expressed genes were assessed based on a negative binomial distribution. Hypergeometric testing was conducted for enrichment analysis.

Figure S10

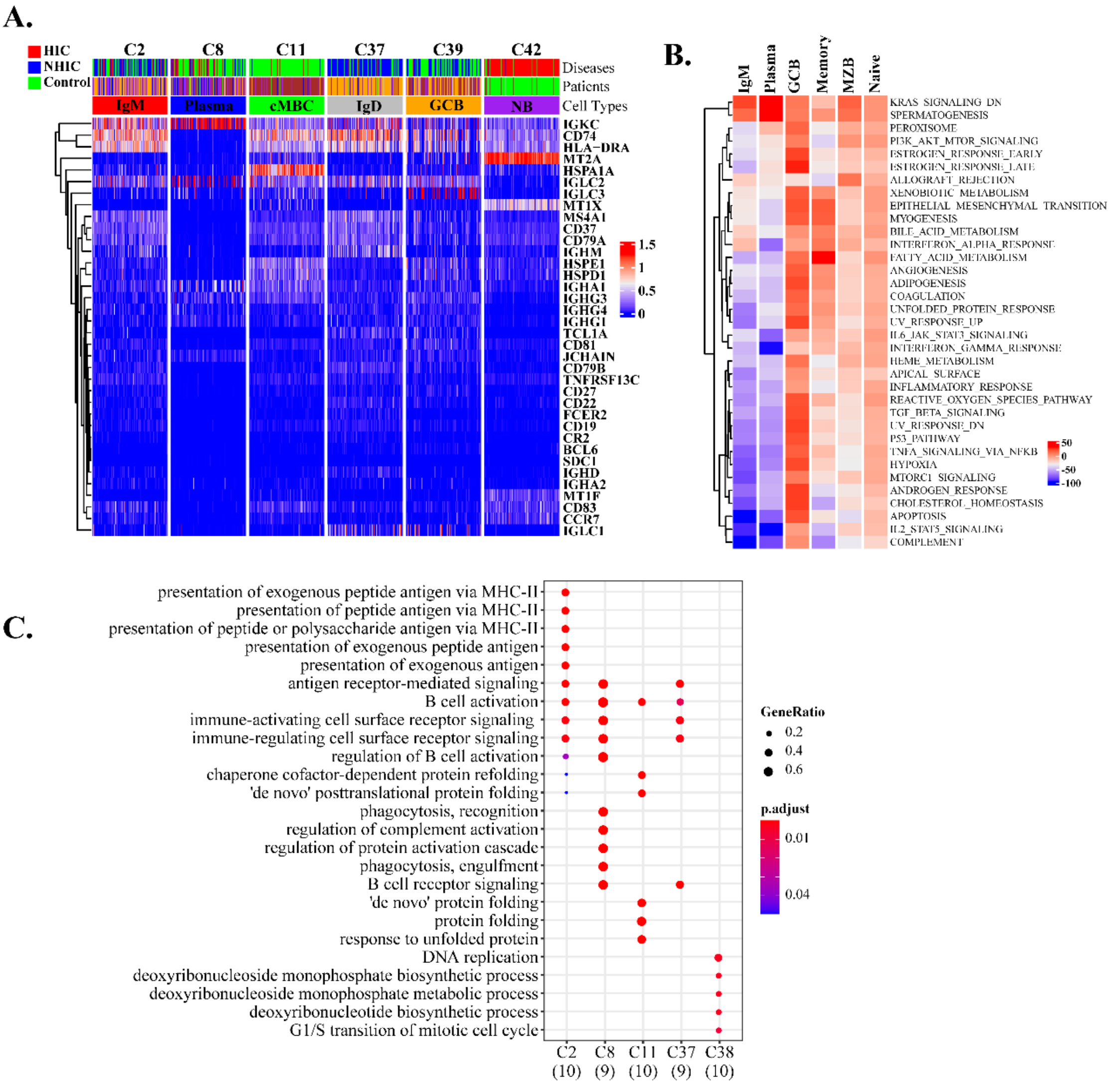

Figure S10:

Functional analysis of differentially expressed genes from different B cells

A. Heatmap of top 10 DEGs of different cell types

B. Heatmap shows difference in pathway activities of hallmark pathways scored by GSVA among different clusters of B cells. Shown are *t*-values from a lineal model.

C. Enriched Gene Ontology (GO) biological processes of upregulated genes in different clusters. Enrichment ratio was computed as the ratio of the observed gene count to background gene count.

Figure S11

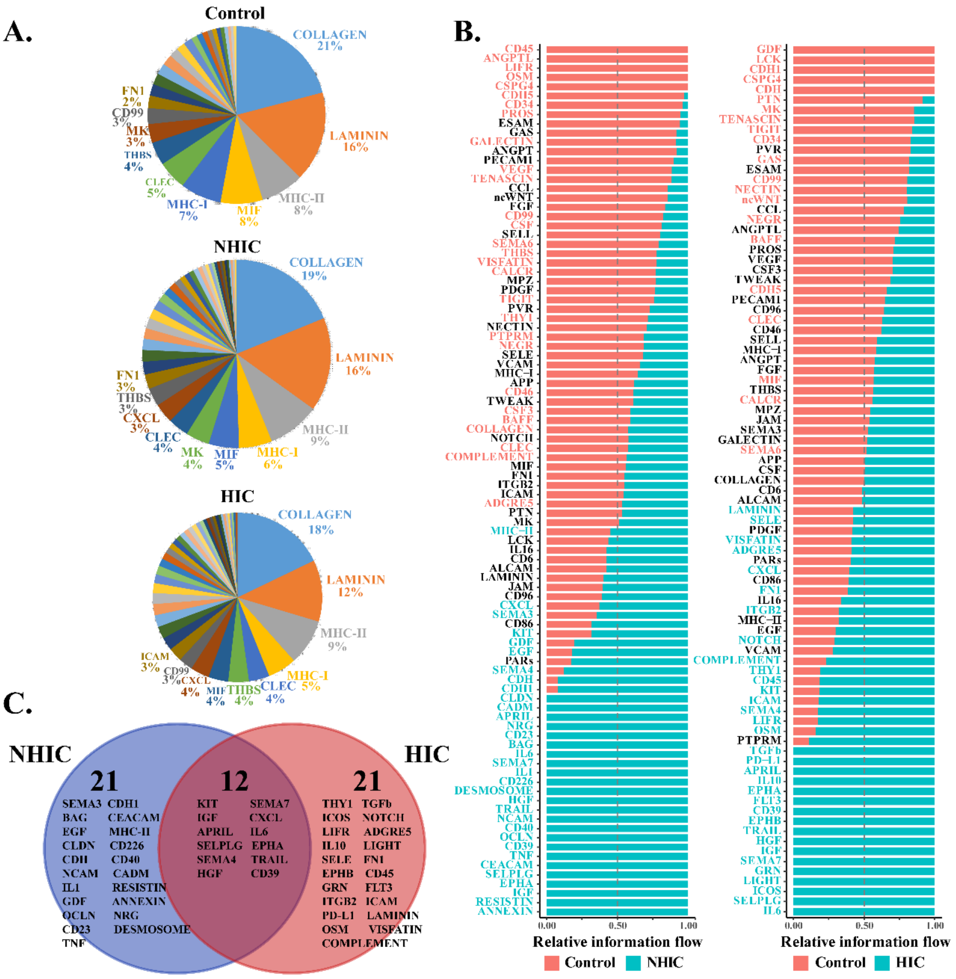

Figure S11:

Analysis of signaling pathways involved in the cell-cell interaction.

A. Pie charts displays the percentages of top 10 signaling pathways from unaffected control, NHIC and HIC.

B. All significant signaling pathways were ranked based on their differences of overall information flow within the inferred networks between Control and NHIC (left), between Control and HIC (right).

C. Venn diagram showing the relationship of significant signaling pathways. Numbers in parentheses refer to the number of pathways found in each class. A detailed list of all the genes is given in the figure.
